# Review of Antimicrobial Properties of Carbon Nanomaterials

**DOI:** 10.3390/ijms27104529

**Published:** 2026-05-18

**Authors:** Lev R. Sizov, Dmitriy A. Serov, Valeriy A. Kozlov, Valery A. Karpov, Fatikh M. Yanbaev, Sergey V. Gudkov

**Affiliations:** 1 Prokhorov General Physics Institute of the Russian Academy of Sciences, Vavilov Str. 38, 119991 Moscow, Russia; leo.sizoff@yandex.ru (L.R.S.); dmitriy_serov_91@mail.ru (D.A.S.); v.kozlov@hotmail.com (V.A.K.); 2 Severtsov Institute of Ecology and Evolution Problems of the Russian Academy of Sciences, Leninsky Prospekt 33, 119071 Moscow, Russia; 3 Federal Research Center ”Kazan Scientific Center of the Russian Academy of Sciences”, ul. Lobachevskogo 2/31, 420088 Kazan, Russia; 4 Department of Fundamental Sciences, Bauman Moscow State Technical University, 5 2nd Baumanskaya St., 105005 Moscow, Russia

**Keywords:** carbon nanomaterials, fullerenes, nanodiamonds, graphene oxide, carbon nanotubes, carbon dots, antimicrobial activity, antibacterial activity, antifungal activity

## Abstract

In various areas of human activity, there is a need for new antimicrobial agents that are minimally hazardous to humans and the environment while remaining effective against multidrug-resistant microorganisms. The use of nanomaterials, particularly carbon-based ones, for this purpose is attracting growing interest. This review presents a quantitative analysis, based on published data, of the antibacterial and antifungal activity of various carbon nanomaterials, focusing on fullerenes, nanodiamonds, graphene oxide, carbon nanotubes, and carbon dots. Their antimicrobial activity is compared both among themselves and with other antimicrobial agents; the effects of their physicochemical properties, functionalization, and photodynamic activity on this activity are also examined.

## 1. Introduction

The pervasive presence of microbes in the human environment constantly impedes anthropogenic activities, thereby creating a persistent demand for novel antimicrobial agents, a need that has been critically exacerbated by the widespread dissemination of antimicrobial-resistant strains [1]. This need goes beyond clinical practice [2] and turns out to be necessary for various fields of anthropogenic activity, including environmental health [3], the woodworking industry [4], the food industry [5], and agriculture [6].

For instance, physical methods of water disinfection—such as ozonation and ultraviolet irradiation—lack a prolonged bactericidal effect [7], whereas chlorination can cause material corrosion and the formation of organochlorine compounds hazardous to health [8]. It has also been shown that bacteria can adapt to chlorine [9]. In food production, the most common method currently used for disinfecting work surfaces is the application of chemical disinfectants such as hypochlorites, hydrogen peroxide, and carboxylic acids. However, sanitization using chemicals requires a complete shutdown of equipment during treatment, and it has been shown to be potentially ineffective for removing biofilm. Furthermore, aggressive chemicals can cause corrosion of steel equipment surfaces, reducing their effective service life [10].

In medicine, antimicrobial agents are required to be non-toxic to humans; this is achieved, in particular, by their effect on molecular targets unique to specific microorganisms. For example, β-lactam antibiotics bind to penicillin-binding proteins involved in the formation of peptidoglycan, consequently disrupting cell wall synthesis [11]. Antimicrobial resistance in individual cells arises due to genetic mutations, which are transmitted within the population through proliferation or horizontal gene transfer, with the latter capable of occurring even between different species. The mechanisms of antibiotic resistance are as follows: enzymatic degradation of the antibiotics; alteration of the antibiotics’ target site; increased active efflux of the drug; reduced cell permeability to antibiotics; and metabolic alterations that allow the cell to function without the damaged target or to overproduce a competitive inhibitor of the antibiotic [12,13]. Additionally, antibiotic resistance is enhanced during bacterial swarming and the formation of biofilms [14]. In biofilms, antibiotics are retained in the extracellular matrix, the exchange of antibiotic resistance genes is facilitated, and signal transmission via quorum sensing regulates gene expression for stress resistance, all while specialized persister cells with decreased metabolic activity ensure the population’s survival against antibiotics [12,13]. In the battle against multidrug-resistant infectious diseases, several novel antimicrobial approaches are under investigation, including bacteriophages, quorum sensing inhibitors, antimicrobial peptides, and synthetic retinoids. Researchers are also exploring drug repurposing, which involves using existing medications, such as anti-inflammatory drugs, for new therapeutic purposes [15].

The fight against the excessive proliferation of microbes in various settings—within the human body, in wastewater, or on industrial production sites—is united under the interdisciplinary One Health approach [16]. Inorganic nanoparticles and nanomaterials (based on metals, metal oxides, or non-metals such as selenium, carbon, and silicon) are capable of acting as antimicrobial agents, with potential applications in various fields of human activity [17,18]. Unlike antibiotics and other antimicrobial agents with a specific molecular mechanism of action, inorganic nanomaterials have a broader spectrum of action [19,20]. In the case of applying inorganic nanomaterials, there is a lower probability of resistance selection in the microbial population, a phenomenon primarily explained by their multidirectional toxic effect against microbes [12]. Among the mechanisms of their antibacterial activity, there can also be a mechanobactericidal effect, to which it is more difficult for bacteria to adapt than to the selective inhibition of targets [21]. Furthermore, inorganic nanomaterials are generally more resistant to the enzymatic activity of bacteria, although there is evidence of the ability of bacteria to degrade them [22]. Unlike aggressive disinfectants like chlorine-containing substances, inorganic nanomaterials are less dangerous to humans and the environment [23,24] and non-corrosive to materials [25,26]. Moreover, nanoparticles can be immobilized on surfaces or injected into coatings for prolonged bactericidal effect and antifouling effect [27,28].

Carbon nanomaterials are of great interest among inorganic nanoparticles in the context of their use as antibacterial agents. They may have membranotropic properties which allow them to penetrate bacterial membranes more easily due to their higher lipophilicity [29] compared to metal nanoparticles [30,31]. Another advantage over metallic nanoparticles is the absence of ion toxicity of carbon nanoparticles [32], since, for example, in the case of Ag nanoparticles, it is the Ag^+^ ions they emit that are considered the most toxic against not only bacteria [33], but also human cells [34,35].

**Figure 1:**
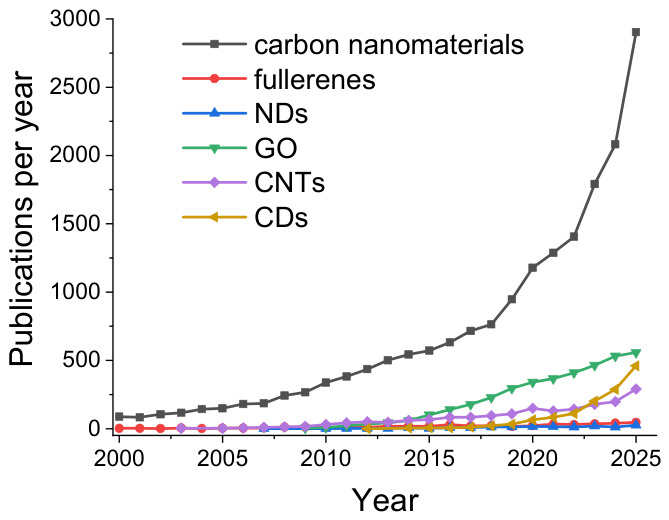
Dynamics of the number of publications retrieved from the OpenAlex database https://openalex.org/ (accessed on 19 March 2026). Search keywords: “carbon”, “fullerenes”, “nanodiamonds”, “graphene oxide”, “carbon nanotubes”, “carbon dots” combined with “(antimicrobial OR antibacterial OR antifungal)”.

Publication trends in the OpenAlex database (Figure 1) show that mentions of the antimicrobial activity of carbon nanomaterials in scientific publications have been increasing rapidly over the past 25 years, with nearly 3000 publications in 2025 for the query “carbon (antimicrobial OR antibacterial OR antifungal)”. Over the last five years, there has been a clear increase in interest in the antimicrobial activity of carbon dots, with their share among the total mentions of all five material types rising from about 15% in 2021 to over 30% in 2025. Graphene oxide is mentioned in the largest total number of publications, and the annual number of publications mentioning its antimicrobial activity is growing steadily. Regarding the antimicrobial activity of carbon nanotubes, hundreds of works have also been published in recent years. A smaller number of publications address the antimicrobial activity of fullerenes (tens per year), and even fewer concern nanodiamonds.

**Figure 2:**
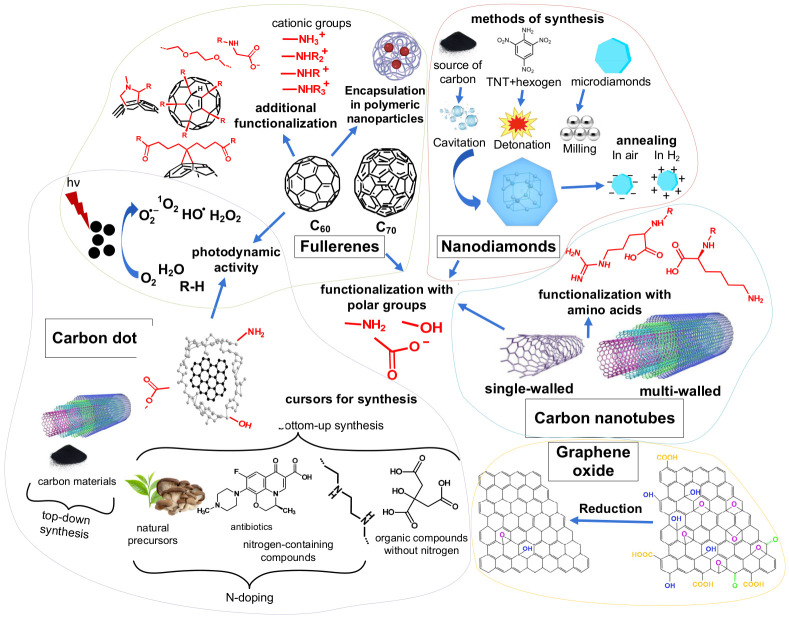
Scheme of analyzed carbon nanomaterials, methods of their synthesis and modification. TNT—trinitrotoluene.

Over the past 5 years, a number of reviews have been published on the antimicrobial (especially antibacterial) activity of both individual types of carbon nanomaterials [36,37,38] and carbon nanomaterials in general [39,40,41,42,43,44,45]. Carbon nanomaterials are united by a single element base, as well as by the significant role that physical interaction with microorganisms plays in their antimicrobial effect [32], which is effective due to their high surface area-to-volume ratio [17]. A special feature of this review is an attempt to conduct a quantitative analysis of the antibacterial and antifungal activity of different types of carbon nanomaterials, similar to what was done previously for other inorganic nanomaterials (titanium dioxide [46], zinc oxide [47], silver oxide [48], selenium [49], copper oxide [50] and aluminum oxide nanoparticles [51]). When comparing the antimicrobial activity of different types of carbon nanomaterials, focus was placed on fullerenes, nanodiamonds (NDs), graphene oxide (GO), carbon nanotubes (CNTs), and carbon dots (CDs) (Figure 2), since antimicrobial activity is most frequently reported and investigated for these types. The influence of various properties (size, charge, structure), functionalization, and synthesis features on their antimicrobial activity is also considered for these nanomaterials.

## 2. Criteria for Data Selection

A systematic search of the academic literature was conducted primarily via the scientific search engine Google Scholar using the following search terms: “fullerenes“, “nanodiamonds“, “graphene oxide“, “carbon nanotubes“, “carbon dots“, “nanobiochar“, “antimicrobial“, “antibacterial“, and “antifungal“. From thousands of publications on carbon nanomaterials, 78 studies were selected for quantitative analysis, and the data from these studies are presented in Table 1. The selection of studies was based not so much on the novelty of the publication as on the availability of the data required for the analysis. This section briefly covers the diversity of the carbon nanomaterials analyzed, their physicochemical properties, and the methods for assessing their antimicrobial activity. The collected data from the studies were categorized and summarized in the form of a table (Table 1). At the end of this section, the criteria for selecting these data and the structure of the table are discussed.

### 2.1. Diversity of Carbon Nanomaterials

**Figure 3:**
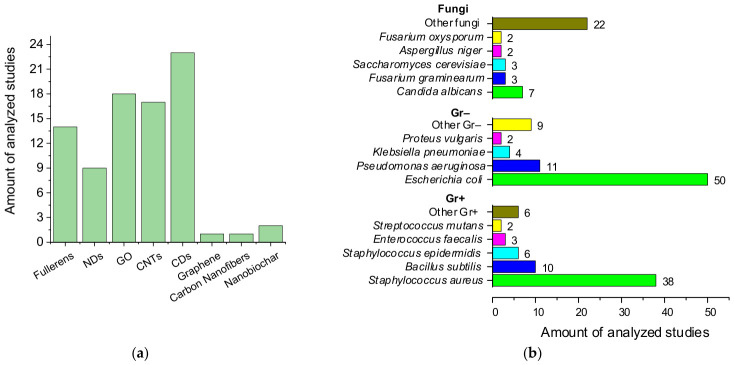
Categorization of the analyzed studies on the antimicrobial activity of carbon nanomaterials by nanomaterial type (**a**) and by microorganism (**b**).

Types of carbon nanomaterials can include both individual allotropes (fullerenes, CNTs, graphite, diamond, graphene [52]) and examples of dimensional (NDs [53]) and chemical (GO, graphite oxide [38]) modifications of known allotropes, as well as nanomaterials characterized by the presence of different structural domains or hybrid allotropic structures (CDs [37]). Figure 3a shows the number of analyzed publications devoted to the study of antimicrobial activity for each type of carbon nanomaterials. For fullerenes, GO, CNTs, and CDs, at least ten articles were selected for analysis, while for NDs, nine articles were selected due to their smaller overall number. It is worth noting that a number of publications provide data on two or more types [29,54,55].

The spatial classification of carbon nanomaterials is based on the number of spatial dimensions in which the nanoscale (<100 nm) is maintained [56]. There are 0D (fullerenes, NDs, CDs, onion-like carbons), 1D (CNTs, carbon nanofibers), and 2D (GO, graphene) nanomaterials [56], for which the nanoscale is maintained in three, two, and one dimension(s), respectively. Although the dimensions of 3D nanomaterials extend beyond the nanoscale, they contain nanostructural elements or domains within their bulk structure (nanobiochar [57], diamond-like carbon [58]).

### 2.2. Physico-Chemical Properties of Carbon Nanomaterials

Physico-chemical properties affect the antimicrobial activity of carbon nanomaterials, and their proper accounting is an important issue [59]. In general, the antimicrobial effect of nanomaterials increases with decreasing size, as their surface area to volume ratio and contact area with bacterial cells increase [17,60]. The sizes of nanomaterials are usually estimated by dynamic light scattering (DLS) [61], electron microscopy [62], and atomic force microscopy [63].

Zeta-potential is a complex function of both the nature of the particles themselves and the properties and concentration of other ions in the solution, pH, temperature, ionic strength, and other factors [64]. The cell walls of microbes are generally negatively charged under physiological conditions, which enables electrostatic interactions with nanomaterials depending on their zeta-potential [65,66,67]. Zeta-potential is usually measured using electrophoretic light scattering [67,68,69] and electrophoresis [70]. It is worth noting that, in some analyzed studies, size and zeta-potential measurements using light scattering were performed in the same medium in which the antimicrobial activity of the nanomaterials was tested [54,68,71], while in others, they were not [72,73], even though both parameters may vary depending on the medium [54,71].

Furthermore, some carbon nanomaterials are capable of antimicrobial activity via photodynamic action, whose efficiency is largely determined by such physico-chemical properties as the quantum yield of the excited triplet state and the singlet oxygen quantum yield [65]. This toxic action mechanism is discussed in more detail in Section 4.1.

### 2.3. Microorganisms Under Study

This review examines the toxic effects of carbon nanomaterials against Gram-negative (Gr−) and Gram-positive (Gr+) bacteria, as well as fungi and fungus-like microorganisms (Table 1). The number of analyzed publications devoted to specific microbial taxa is shown in Figure 3b. The largest number of publications focused on bacteria: 76 articles on 10 species of Gr− bacteria and 65 articles on 11 species of Gr+ bacteria, with the typical species *Escherichia coli* (50 articles) and *Staphylococcus aureus* (38 articles) predominating, respectively. For Gr−, other encountered species are *Pseudomonas aeruginosa*, *Klebsiella pneumoniae*, *Proteus vulgaris*, *Ralstonia solanacearum*, *Salmonella* sp., *S. enterica* (including serovars *Salmonella typhimurium* and *Salmonella* Paratyphi β), *Shigella flexneri*, *Acinetobacter baumannii,* and *Methylobacterium* sp. For Gr+, other encountered species are *Bacillus subtilis*, *B. anthracis*, *S. epidermidis*, *Enterococcus faecalis*, *Streptococcus mutans*, *Propionibacterium acnes*, *Listeria monocytogenes*, *Lactobacillus acidophilus*, *Bifidobacterium adolescentis* and *Micrococcus luteus.* For fungi, 39 studies on 26 species were analyzed, with the greatest number of studies concerning the yeast *Candida albicans* (7 articles); other encountered species are *Candida* sp., *C. tropical*, *Saccharomyces cerevisiae*, *Fusarium graminearum*, *F. oxysporum*, *F. culmorum*, *F. poae*, *Aspergillus niger*, *A. flavus*, *A. fumigatus*, *A. oryzae*, *Penicillium chrysogenum*, *P. italicum*, *P. lilacinum*, *Trichophyton mentagrophytes*, *T. rubrum*, *Alternaria alternata*, *Bipolaris sorokiniana*, *Botrytis cinerea*, *Cladosporium cladosporioides*, *Coriolus versicolor*, *Gloeophyllum trabeum*, *Pyricularia grisea*, *Rhizoctonia solani*, and *Microsporum canis*. One publication each was found on the antimicrobial activity of carbon nanomaterials against the oomycetes *Phytophthora infestans* and *Pseudoperonospora cubensis*. Unlike bacteria, studies on antifungal activity are more often conducted within specific contexts such as the wood processing industry [4] and agriculture [6,74,75], which accounts for the greater number of species encountered.

### 2.4. Microbiological Parameters for Antimicrobial Activity Assessment

Antimicrobial activity is categorized into biostatic (bacteriostatic/fungistatic) activity, which halts microbial reproduction, and biocidal (bactericidal/fungicidal) activity, which additionally reduces the microbial count [76]. Biocidal is the more desirable effect [77] and is typically achieved simply by increasing the concentration of the antimicrobial agent [78], although the mechanism of some antibiotics may involve only the inhibition of reproduction [76].

**Figure 4:**
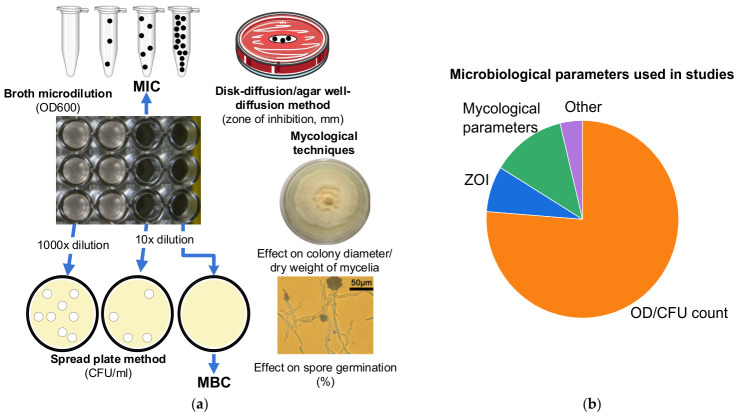
Methodology scheme for studying the antimicrobial activity of compounds and nanomaterials (**a**). Categorization of the analyzed studies on the antimicrobial activity of carbon nanomaterials by the used microbiological parameters (**b**).

The determination of bacteriostatic and bactericidal activity is guided by the Clinical and Laboratory Standards Institute (CLSI) Performance Standards for Antimicrobial Susceptibility Testing [79]. The principles of the methodology are illustrated in Figure 4a. The indicator of bacteriostatic or fungistatic effect is the minimal inhibitory concentration (MIC), at which the antimicrobial agent prevents the development of turbidity in a previously clear suspension of 10^5^–10^6^ cells/mL during overnight incubation [80] (Figure 4a shows, as an example, a row of culture plate wells with a clear liquid). The absence of turbidity is determined by the naked eye or by measuring the optical density at 600 nm (OD) using an appropriate instrument [73]. The broth dilution technique [73,81,82,83] (or a similar technique using dilution in salt solution [55,68] or simply in water [84]) was predominantly used in the analyzed studies to obtain samples containing microorganisms with nanomaterials at various concentrations. As shown in Figure 4a, nanoparticle concentrations exceeding the MIC generally no longer alter the optical density, unless the nanoparticles themselves contribute to this parameter. The agar dilution method can also be used to prepare a series of concentrations [85].

Subsequently, according to the standard protocol, to determine the bactericidal effect, it is necessary to demonstrate that the microorganisms are unable to recover after exposure to the nanomaterial at the MIC level or higher [80]. For colony counting [86], the spread plate method [87] is used, in which a drop of the microbial suspension is evenly distributed onto prepared solid agar in a Petri dish. For each sample, tenfold dilutions [68] are prepared before plating to achieve a suitable colony count range (approximately 30–300 colonies per plate) [88]; these dilutions may differ between samples, as shown in Figure 4a. When converting the average colony count per plate to colony-forming units per milliliter (CFU/mL), the use of different serial dilutions for the control and the sample containing the antimicrobial agent results in a reduction in the microbial count by a factor of 100, 1000, or more [65,89]. The indicator of bactericidal activity is the minimal bactericidal concentration (MBC) at which no colonies are observed on the plates [83] or the microbial count is reduced by 99.9% [80,81,90] (Figure 4a schematically shows a Petri dish with no colonies). Additionally, the bactericidal agent is determined by the MBC/MIC ratio between 1 and 2, as recommended by CLSI [82], or no more than 4, as suggested in other studies [77,78].

In some of the analyzed publications, the MIC was interpreted differently. For example, in [67], the MIC was taken as the concentration causing a 90% reduction in CFU, whereas it can also be defined as the minimum concentration at which no cellular reduction in MTT [91] or resazurin [92] is detected, although these assays are typically used to assess metabolic activity rather than bacterial viability [93,94]. Overall, even in experiments conducted according to CLSI standards, a certain heterogeneity of conditions exists. For example, for *E. coli* CFU counting, different incubation times (1 h [95] and 24 h [84]) and different initial cell suspension concentrations (10^5^ [90], 10^7^ [78], and 2 × 10^8^ CFU/mL [95]) are encountered.

Antimicrobial activity is also assessed using other methods. In addition to the aforementioned MTT and resazurin assays, the analyzed publications employed the disk-diffusion method [96] and the well-diffusion method [92,97] with the zone of inhibition (ZOI) as the endpoint, fluorescence microscopy with specific dyes [98], optical microscopy [75], as well as techniques specific to antifungal activity determination, in which the effect of the antifungal agent on colony diameter [99], mycelial dry weight [100], and spore germination rate [101] is assessed (Figure 4a). Furthermore, the mechanism of action against microorganisms is elucidated using electron microscopy [98,99,100,101]. It is worth noting that the CFU counting method, most commonly used to determine MBC, may not reflect a reduction in the number of viable microbes, but rather a reduction in their colony-forming ability [95]. Confirmation of biocidal activity requires images of dead cells obtained using the same electron microscopy technique [62], the use of propidium iodide [78] or dyes with similar functionality [83,102], or measurement of the leaked nucleic acids [103].

Two-thirds of the analyzed studies used standard methods with OD and CFU count as the microbiological parameters to assess antimicrobial activity (Figure 4b). In 12.5% of the studies, the antimicrobial activity of nanomaterials was assessed using mycological parameters, in 7.5% using ZOI, and in the remaining small proportion using other microbiological parameters.

### 2.5. Information About Data Selection and Presentation

Studies and the data obtained from them were selected according to all the categories discussed above: by type of carbon nanomaterial, their physicochemical properties, studied microorganisms, and microbiological methods. Thus, in the carbon nanomaterials under consideration, only a carbon atom serves as the structural basis; for this reason, data on graphitic carbon nitride (g-C_3_N_4_) [104] are not included in this review, since nitrogen is also a major element in its structural lattice. In addition to data on the main five types of carbon nanomaterials (fullerenes, NDs, GO, CNTs, and CDs), Table 1 shows data from several articles on graphene, carbon nanofibers, and nanobiochar (Figure 3a). In addition, other carbon nanomaterials are mentioned in Section 3.6.

Furthermore, the tetravalency of the carbon atom not only accounts for the diversity of carbon nanomaterial types, but also for the variety of ways in which they can be modified [105]. Figure 2 presents the varieties and modifications of nanomaterials considered in this review and discusses them in more detail in Section 3. For the quantitative analysis of the antimicrobial activity of the carbon nanomaterials themselves, data from the studies were included only if the nanomaterials were not combined with antibiotics [106,107], silver nanoparticles [108,109,110,111,112], or other antimicrobial agents whose activity overlaps with that of the carbon nanomaterials. Only functionalization aimed at enhancing water dispersibility and/or modifying the surface charge of the nanomaterial was included in this analysis, but without increasing cell affinity through antibodies [113] or other ways of active targeting [114]. The use of nanomaterials in antimicrobial photodynamic therapy (aPDT) was analyzed, but without the use of the far-ultraviolet range [78], which itself is a disinfection method [7], and also without photothermal therapy [104,115]. The antimicrobial activity of nanomaterials was considered primarily in suspension form; some data obtained using the disk-diffusion or well-diffusion method were included, but data on the activity of nanomaterials incorporated into solid [30,116] or gel-based [117] composites were excluded, as the matrix itself may contribute to the antimicrobial effect.

The data in Table 1 are organized first by nanomaterial type, then by individual publication within each type, and finally by variant of the same nanomaterial type within each publication. Within a publication, separate rows for the same nanomaterial variant may differ with respect to its application in antimicrobial photodynamic therapy, the microorganism species tested, or the microbiological parameter used. The term “microbiological parameters” refers to the indicators that reflect the method employed: OD (assessment of culture growth in suspension by turbidity); CFU count (spread plate method); ZOI (disk-diffusion and well-diffusion methods); mycological parameters (determination of colony diameter, mycelial dry weight, and spore germination rate); fluorescence intensity of propidium iodide and SYTO9; images of damaged cells obtained by optical microscopy; and MTT staining intensity.

The “Size” column reports diameter values for 0D nanomaterials, length for 1D nanomaterials, and lateral size for 2D nanomaterials. When selecting size data, hydrodynamic diameter values were avoided for 1D and 2D nanomaterials, since the conventional DLS method is not appropriate for sizing non-spherical particles [118]. For 0D nanomaterials, DLS data were used only for particles with a hydrodynamic diameter of 10 nm or larger, owing to the detection limits of the method [119]. In general, when multiple methods were used to determine size, preference was given to data obtained by transmission electron microscopy (TEM), scanning electron microscopy (SEM) or atomic force microscopy [63,68].

In the analysis, toxicity toward individual strains, subspecies, or serovars was not distinguished. If the work contained data on multiple strains, only one was selected [99]. Data on antibiotic-resistant strains were excluded if the publication contained data on non-resistant strains [120], or were included as data for the species as a whole [67]. The toxicity of nanomaterials toward swarming cells, biofilms, and microbial communities was not examined.

Table 1 presents experimental data from studies that both identified and did not identify antimicrobial activity. Antimicrobial activity data are not categorized as biostatic or biocidal. Overall, these two types of activity overlap, and most antimicrobial agents exhibit both [77]. Following a similar principle, Table 1 lists nanomaterial concentrations without reference to MIC or MBC; not all publications used these concepts, nor did the observed effect always fall within these definitions in every case [29,62,103,109]. The more general term “inhibitory concentrations” is used instead of this. In addition, concentrations at which no antimicrobial effect was observed are also indicated. Non-toxic concentrations are considered separately from those with an antimicrobial effect, as they do not represent threshold values but are nevertheless of certain interest. Concentration values are given in mg/mL, with conversion from µM [121], µg/mL [29], and ppm [109] performed where necessary.

The distributions of inhibitory concentrations are described by the interquartile range (between Q1 and Q3), representing the range of the 50% most typical values in the dataset [122]. In the figures, data distributions are visualized as box plots with overlaid individual data points, where each point corresponds to a single experimentally published datum (Table 1). The box plots show the interquartile range (box), median (center line), and minimum and maximum values (whiskers) of the distributions. Nonparametric methods were used to compare inhibitory concentration values across different nanomaterials or microorganisms: the Mann–Whitney U test for two groups, and the Kruskal–Wallis test followed by Dunn’s test for pairwise comparisons for multiple groups. A statistically significant difference was considered at *p* < 0.01, although differences at *p* < 0.05 are also marked in the figures.

For the majority of concentrations exhibiting antimicrobial activity, Table 1 also reports the inhibition rate, defined as the percentage change in the measured microbiological parameter (OD, CFU count, ZOI, spore germination rate, or other) in a given experiment relative to the control. The concentrations included are those reported as MIC or MBC, or those approximating these values; consequently, most inhibition rate values are 100% or close to it. In some studies, the inhibition rate of the nanomaterials was markedly lower, but only rates of 10% or higher were included. In the figures, the inhibition rate is depicted using a color scale. For concentrations where antimicrobial activity was assessed by disk-diffusion or well-diffusion methods (measuring ZOI) or by optical microscopy, inhibition rate values were not calculated, which is represented by the color black in the figures.

## 3. Antimicrobial Activity of Different Types of Carbon Nanomaterials

### 3.1. Fullerenes

Figure 2 shows the preparation and application variants of fullerenes examined in this review. Among fullerenes, C_60_ is most commonly tested for antimicrobial activity [55,100,119,123,124], whereas C_70_ is tested much less frequently [67,89,125,126]. Higher-order fullerenes are challenging to synthesize [127], and their biological properties remain largely unexplored [128]. Unlike other carbon nanomaterials, fullerenes exist in a molecular form [129]. In aqueous solution, they form aggregates with sizes, some [130] exceeding the 100 nm nanoscale range [56]. Analysis of the collected data revealed no correlation between fullerene nanoparticle size and inhibitory concentration values.

In fullerenes, carbon is sp^2^-hybridized, and the numerous double bonds allow for chemical modification [36]. While methods for preparing aqueous suspensions of pristine fullerenes are available [119], the stability of such suspensions is improved by attaching solubilizing groups (amino [106], carboxyl [125], and hydroxyl [121] groups) directly to the cage [131,132] or via cyclopropane [89], pyrrolidine [65], or other derivatives [36,106]. This review also covers fullerenes solubilized with polyvinylpyrrolidone polymer nanoparticles [29,119]. Other approaches for obtaining stable aqueous suspensions of fullerenes have also been reported [131].

**Figure 5:**
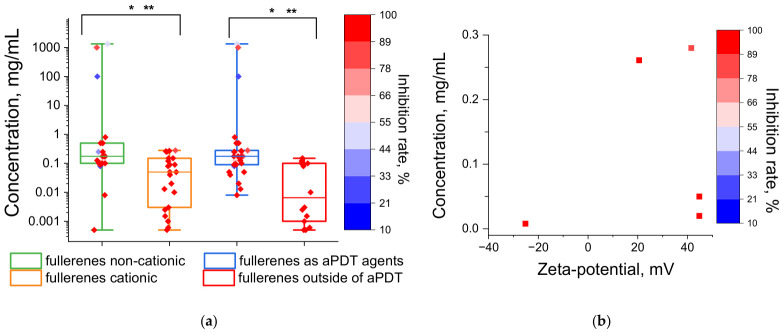
Inhibitory concentration values for cationic and non-cationic fullerenes, with and without the application of aPDT (upon light excitation) (**a**); Mann–Whitney comparisons have been made (*—*p* < 0.05, **—*p* < 0.01). Dependence of the inhibitory concentrations of fullerenes on the zeta-potential (**b**). The color scale shows the inhibition rate (%) at the given inhibitory concentration.

Another common modification strategy for fullerenes is to impart a positive charge via amine nitrogen [65,67,70,89,106,133] (Figure 2), which increases their affinity for negatively charged microbial cell walls [65,66,67]. A statistically significant difference was found when comparing the antimicrobial concentration values of cationic and non-cationic fullerenes, demonstrating that cationic fullerenes are more toxic to microbes (Figure 5a). The interquartile ranges for cationic and non-cationic fullerenes were 0.00288–0.15 and 0.1–0.5 mg/mL, respectively; furthermore, all inhibition rate values for cationic fullerenes exceeded 75%, unlike those for non-cationic fullerenes (Figure 5a). The scatter plot shows that inhibitory concentrations within the typical range are achieved mainly at positive zeta-potential values (Figure 5b). The only data point with a low inhibitory concentration and a negative zeta-potential corresponds to a glycine–fullerene conjugate [73]. The amine nitrogen of the glycine residue may still promote affinity for the bacterial wall, even in the absence of a positive charge, for example, via hydrogen bonding with the phosphate oxygen of teichoic acids [134].

An even stronger enhancement of antimicrobial activity is seen for the photodynamic effect (application of aPDT). A statistically significant difference was observed between the inhibitory concentration values with fullerene irradiation (interquartile range 0.0009–0.1 mg/mL) and without irradiation (interquartile range 0.09–0.28 mg/mL) (Figure 5a). When excited by photons in the ultraviolet and visible (blue/green) spectral ranges, fullerenes are highly efficient at generating the highly toxic singlet oxygen (Type II mechanism of photodynamic action), owing to the high (up to 100%) quantum yield of the triplet state production [36]. In addition, fullerenes can photogenerate reactive oxygen species (ROS) via a Type I mechanism, leading to the formation of superoxide anion radical, hydrogen peroxide, and hydroxyl radical [36].

### 3.2. Nanodiamonds

The synthesis methods and preparation procedures for nanodiamonds analyzed in this review are shown in Figure 2. The review focuses mainly on detonation nanodiamonds, the most common type of which are synthesized (including on an industrial scale [135]) by the detonation decomposition of explosives (a mixture of trinitrotoluene with hexogen or others explosives [93]) under oxygen-deficient conditions [72]. Detonation nanodiamonds have a diameter of approximately 5 nm, which creates a large surface-to-volume ratio [93]. Their core possesses a diamond structure with sp^3^-hybridization and is surrounded by a partially oxidized graphitized layer containing sp^2^-hybridized carbon [72].

A distinctive feature of non-detonation nanodiamonds—including milled NDs [93] and those produced by hydrodynamic [135] or ultrasound [97] cavitation—is that they can be endowed with stable fluorescent properties using heteroatoms [93]. Pronounced antimicrobial toxicity was observed for NDs obtained by ultrasound treatment of graphite oxide [97]. In the study by Ong et al., NDs were produced by milling synthesized bulk diamond, yielding more heterogeneous and larger nanoparticles (tens of nanometers in diameter) than detonation NDs [93]. These NDs are not toxic to bacteria, but have a high affinity for *S. aureus* cells, thereby reducing the bacteria’s colony-forming ability [95].

**Figure 6:**
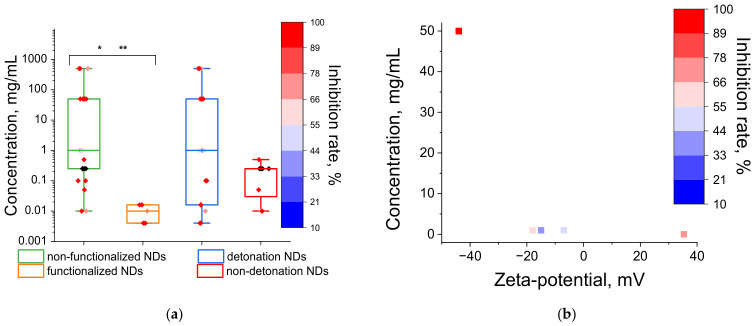
Inhibitory concentration values for functionalized and non-functionalized NDs and detonation and non-detonation NDs (**a**); Mann–Whitney comparisons have been made (*—*p* < 0.05, **—*p* < 0.01). Dependence of the inhibitory concentrations of NDs on the zeta-potential (**b**). The color scale shows the inhibition rate (%) at the given inhibitory concentration. The black color of the symbols indicates that inhibition rate values were not calculated.

No statistically significant difference was observed between the inhibitory concentration datasets for detonation NDs and non-detonation NDs (Figure 6a). Nevertheless, given the absence of true toxicity in milled NDs, detonation NDs can still be presumed to exhibit more pronounced antimicrobial activity. Functionalization of detonation NDs with carboxyl, hydroxyl, and amino groups may also enhance antimicrobial activity (Figure 6a); however, data are available from only two studies [90,136], and the small sample size precludes definitive conclusions.

Annealing procedures exist for stabilizing aqueous suspensions of nanodiamonds: annealing in air yields negatively charged NDs, while annealing in a H_2_ atmosphere yields positively charged NDs [72]. A positive charge may increase the affinity of detonation NDs for microorganisms, but does not confer toxicity [72,136,137]. Comparison of inhibitory concentrations and zeta-potential of NDs from the analyzed publications reveals no correlation (Figure 6b). Wehling et al. attribute the toxicity of NDs toward microbes to the composition and distribution of oxygen-containing functional groups, in particular, carboxylic acid groups in acid anhydride forms [72]. The study by Khanal et al. found that amination of NDs, compared with carboxylation and hydroxylation, specifically promotes antimicrobial activity against *S. aureus*, with this effect not being attributable to a positive charge—a finding that correlates with the amination of fullerenes discussed above [136].

### 3.3. Graphene Oxide

Graphene oxide is classified as a graphene family nanomaterial consisting of a two-dimensional monolayer of sp^2^-hybridized carbon atoms arranged in a hexagonal structure (Figure 2) [68]. Unlike the parent allotrope graphene, GO possesses a high density of oxygen-containing surface groups (mainly epoxy and hydroxyl groups), generated during the oxidation of graphite monolayers [68], predominantly using the modified Hummers method with sulfuric acid, potassium nitrate, and potassium permanganate [68,78,100,109]. Reduced GO, which has a surface similar to that of graphene (Figure 2), is produced by heating in an inert atmosphere [54], by treatment with reducing agents (hydrazine [62], sodium borohydride [138], or starch [82]), or via bioreduction by fungi [139].

**Figure 7:**
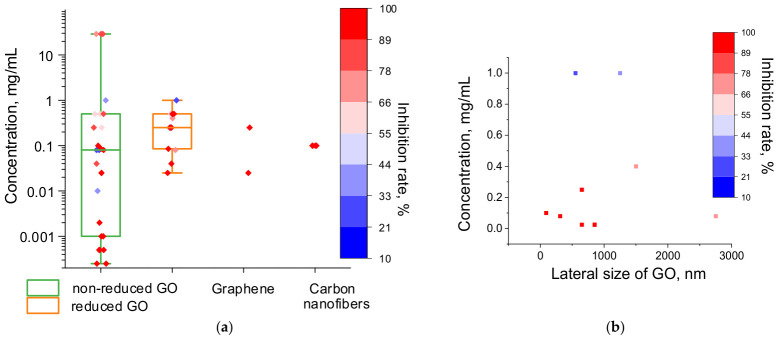
Inhibitory concentration values for reduced and non-reduced GO, graphene and carbon nanofibers (**a**). Dependence of the inhibitory concentrations of GO on the lateral size (**b**). The color scale shows the inhibition rate (%) at the given inhibitory concentration.

Several studies have demonstrated that, upon reduction, GO becomes more hydrophobic and less toxic to Gr− bacteria [54,62,68] and fungi [100]. Comparative analysis of data from various publications shows that reduced GO exhibits slightly lower inhibitory concentration values (interquartile range 0.08–0.5 mg/mL) than non-reduced GO (interquartile range 0.001–0.5 mg/mL), but the differences are not statistically significant (Figure 7a). Akhavan et al. reported higher antibacterial activity for reduced GO compared to GO, but, in that study, the materials were deposited on a stainless steel substrate [140]. Furthermore, increased bacterial toxicity of the reduced form of GO may occur during bioreduction of GO under anaerobic conditions [141].

Although the monolayer thickness of all GO variants is about 1 nm, their lateral size can vary widely, from tens of nanometers [142] to 9000 nm or more [143]. Some studies claim that smaller sizes enhance the antimicrobial activity of GO [142], while others directly oppose this, asserting that large lateral size is beneficial [143]. Based on the data collected in this review, greater toxicity is associated with GO of lateral size < 1000 nm (Figure 7b). However, specific antimicrobial mechanisms are also known for extra-large sheets and are discussed in more detail in Section 4.1.

Due to the large number of oxygen-containing groups on the surface, including carboxyl groups [68,140], the zeta-potential of GO and its reduced form have negative values [54,68,139]; no correlation between these values and the inhibitory concentrations has been found.

### 3.4. Carbon Nanotubes

**Figure 8:**
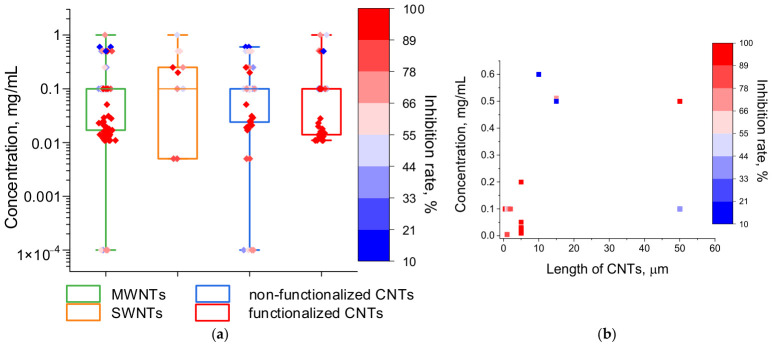
Inhibitory concentration values for MWNTs and SWNTs, functionalized and non-functionalized CNTs (**a**). Dependence of the inhibitory concentrations of CNTs on the length (**b**). The color scale shows the inhibition rate (%) at the given inhibitory concentration.

CNTs are rolled graphene sheets and are divided into single-walled (SWNTs, outer diameter 1–2 nm) and multi-walled (MWNTs, outer diameter up to 100 nm) [39] (Figure 2). Despite reports that SWNTs have greater antimicrobial activity than MWNTs [140,144]; this trend is not evident from the data available (Figure 8a). It is worth noting that all of the data on the non-toxicity of CNTs found relate specifically to MWNTs (Table 1); however, far more data on the antimicrobial activity of MWNTs have also been collected than for SWNTs. Pristine CNTs tend to aggregate and precipitate in water, so functionalization with polar groups is applied to enhance the stability of their aqueous suspensions [84,145,146] (Figure 2). However, based on the available data, functionalized and non-functionalized CNTs show similar toxicity toward microorganisms, at least in in vitro experiments (Figure 8a).

Moskvitina et al. found that shorter CNT length enhances toxicity against bacteria [84]. A similar trend is seen in the data collected for this review when comparing CNT length and inhibitory concentration values (Figure 8b). The lowest inhibitory concentrations (0.005–0.2 mg/mL) are associated with CNTs up to 5 μm in length. For longer CNTs (50 μm), Chen et al. reported several inhibitory concentration values (0.1 mg/mL) in the same range, but with an inhibition rate of only up to 40% [147]. Wang et al. detected no effect of CNT length on the spore germination rate of *Fusarium graminearum* [101]. In that same study, no effect of CNT charge on spore activity was found, although zeta-potential was not measured in that study or in any of the other studies reviewed [101].

### 3.5. Carbon Dots

CDs are a heterogeneous group of quasi-spherical core–shell carbon nanoparticles exhibiting strong photoluminescence and ranging in size up to 10 nm [37] or, in some cases, somewhat larger [61]. Carbon quantum dots are characterized by a structured core with predominantly sp^2^-hybridization and quantum size effects [148]. Graphene quantum dots, which are small fragments of one or several graphene layers, also possess an ordered structure [149]. Carbon nanodots and carbonized polymer dots have an amorphous core with carbon mainly in sp^3^-hybridization; their photoluminescence arises from mechanisms other than quantum confinement [148]. The outer shell of carbon dots contains heteroatom functional groups (hydroxyl, carboxyl, amino, etc.) and is crucial both for photoluminescence and for stabilizing aqueous suspensions [150]; however, carbon remains the primary structural element of CDs, accounting for 80% or more by mass [151].

A wide range of carbon sources can be used to synthesize CDs (Figure 2). In general, synthesis methods are divided into top-down and bottom-up approaches [152]. The top-down approach breaks down carbon nanomaterials (CNTs, fullerenes) and large-sized (bulk) carbon materials (graphite, carbon black) that already possess aromatic motifs as CD precursors using techniques such as laser ablation, ultrasound, or acid treatment [37,148]. Bottom-up methods involve the carbonization and fusion into CDs of organic molecules from specific chemical compounds, polymers, or biomass from natural precursors, for example, via hydrothermal treatment in an autoclave [37,149]. In recent years, the bottom-up approach has become the most commonly used for a number of reasons [148]; in this review, data on top-down CDs (derived from MWNTs) are presented from just one study [6].

**Figure 9:**
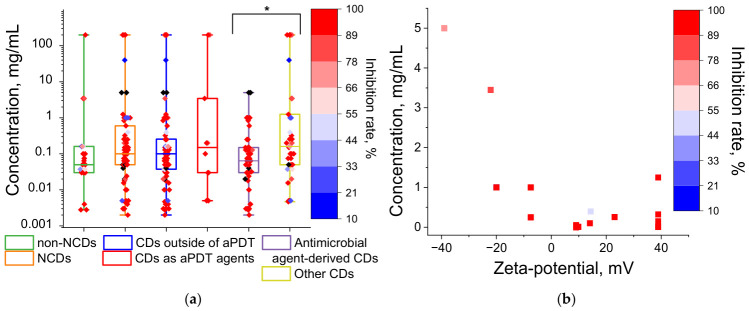
Inhibitory concentration values for nitrogen-doped carbon dots (NCDs) and non-NCDs, CDs with and without the application of aPDT (upon light excitation), antimicrobial agent-derived CDs and CDs synthesized from compounds lacking pronounced or selective toxicity (“other CDs”) (**a**); Mann–Whitney comparisons have been made (*—*p* < 0.05). Dependence of the inhibitory concentrations of CDs on the zeta-potential (**b**). The color scale shows the inhibition rate (%) at the given inhibitory concentration. The black color of the symbols indicates that inhibition rate values were not calculated.

In bottom-up CD synthesis, precursors with proven antimicrobial activity are frequently used, since CDs may retain certain precursor properties—in particular, antimicrobial activity—due to the preservation of active surface functional groups [37,153]. This review covers antimicrobial agent-derived CDs based on specific compounds (ciprofloxacin [154], chlorhexidine gluconate [155], levofloxacin [156], 2-methoxy-1,4-naphthoquinone [85], 2,4-dihydroxybenzoic acid [157]) and natural precursors (extract of the fungus *Diaporthe unshiuensis* [61], oyster mushroom [92], tea and osmanthus leaves [158], turmeric leaves [69], Henna plant [96], *Artemisia argyi* leaves [159], Forsythia [4]) (Table 1). Other CDs include those synthesized from compounds lacking pronounced or selective toxicity (citric acid [160], diethylenetriamine [120], urea [63], sodium fluoride [161], o-phenylenediamine [115]) or from substrates (milk vetch leaves [158], pumpkin seeds [75]) (Table 1). Top-down CDs derived from MWNTs [6] are also included among “other CDs” for comparative analysis; although MWNTs have known antimicrobial properties, their mechanism of action does not involve specific targets, so no inherited functional-group-mediated toxicity can be assumed. Comparison of inhibitory concentrations for antimicrobial agent-derived CDs (interquartile range 0.03–0.15 mg/mL) and other CDs (interquartile range 0.05–1.1 mg/mL) revealed a difference, but with *p* > 0.01 (Figure 9a). Overall, even for antimicrobial agent-derived CDs, non-specific mechanisms—ROS generation, coating, and other mechanisms typical of carbon nanomaterials (discussed in Section 4.1)—likely play a major role in their toxicity, as demonstrated, for example, in [69,153,158].

Nitrogen doping (or doping with other heteroatoms) may enhance the antimicrobial activity of CDs [75], though this effect is not clearly observed in the collected data (Figure 9a). The enhancement of antimicrobial action by nitrogen doping may be attributed to the positive charge imparted to the nanoparticles via amine groups, enabling electrostatic interactions with microbial cell walls [63,155]. A certain trend indicating the importance of positive charge for CD antimicrobial activity is evident from the data collected (Figure 9b).

Nitrogen doping was also employed to optimize the photophysical and photochemical properties of CDs that influence their antimicrobial activity [162], as CDs, like fullerenes, are capable of photodynamic action [163,164], including under visible light excitation [157,165,166]. Data analysis showed no significant impact of the photodynamic effect on CD inhibitory concentration values (Figure 9a). Unlike fullerenes, most synthesized CDs exhibit low quantum yields of the triplet state production and, consequently, for singlet oxygen generation, although examples with singlet oxygen quantum yields of 50% or higher have been reported [166,167]. At low quantum yields, irradiation of CDs either has virtually no effect on toxicity [161] or merely accelerates the antimicrobial effect over time [157].

Sun et al. reported that the antibacterial activity of CDs increases as their size decreases [155]. However, CD sizes generally vary within a fairly narrow range—from 1 to 14 nm among the analyzed samples (Table 1)—and no such correlation was found in the collected data.

### 3.6. Other Carbon Nanomaterials

Graphene, the structural basis of graphene-family nanomaterials, has antibacterial activity, though less pronounced than that of GO [68]. Other sp^2^-hybridized carbon nanomaterials—carbon nanofibers made of graphitic-like planes—also show toxicity against Gr− and Gr+ bacteria comparable to that of CNTs and NDs [84]. Onion-like carbons also consist of graphitic-like planes, but form hollow carbon nanoparticles [56]; Moskvitina et al. found no antibacterial activity for them [84]. Weak antimicrobial activity was also reported for graphite and graphite oxide [62].

In amorphous carbon, atoms lack a defined structure and exhibit sp^2^-/sp^3^- hybridization with fragments of sp-hybridization [168]. Considering amorphous carbon separately from carbon dots (which include the carbon nanodot type based on amorphous carbon [37,148]), it is mainly studied and used as thin films, with a focus on their mechanical and electrochemical properties [168]. Diamond-like carbon structures based on amorphous carbon are also produced as thin films, and their relatively high sp^3^-bond content (40–60% [168]) gives them high hardness [169]. Diamond-like carbon coatings themselves show antifouling and antibacterial properties [170]; however, evidence indicates that this activity is insufficient without additional antibacterial agents [58,171]. Kang et al. showed that purifying MWNTs from amorphous carbon did not affect their antibacterial activity, which indirectly indicates its low toxicity [172]. Conversely, nitrogen-doped amorphous carbon nanosheets exhibited high antibacterial activity exceeding that of GO, and slightly weaker activity was also found for a carbonized carbon material, also based on amorphous carbon [173]. In the study by Gudkov et al., the inhibitory effect on bacteria of amorphous carbon nanoparticles within a polymethyl methacrylate composite was determined [30].

Antimicrobial activity has also been reported for carbon nanoparticles of mixed composition that do not fall under the category of CDs. For instance, nanoparticles isolated from soot can contain amorphous and graphitized carbon [174] and even fullerenes [175], and several studies have demonstrated their ability to inhibit both Gr− and Gr+ bacterial growth [174,175,176]. Nanobiochar comprises porous nanoparticles up to 100 nm in size derived from biochar [177]. Nanobiochar itself is non-toxic to microorganisms and requires the separate loading of antimicrobial agents to achieve antimicrobial activity [44,81,108]. For activated carbon, which can also be obtained from renewable and cheap biomaterials [178], likewise, no antimicrobial activity has been detected [100].

### 3.7. Comparison of Antimicrobial Activity of Different Types of Carbon Nanomaterials

**Figure 10:**
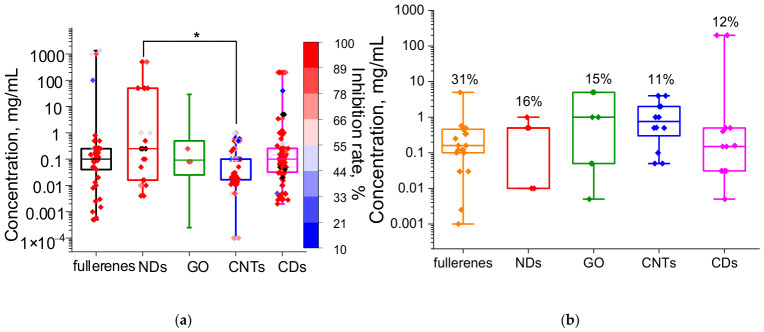
Inhibitory concentrations of fullerenes, NDs, GO, CNTs, and CDs, compared using the Kruskal–Wallis test with pairwise comparisons according to Dunn’s test (*—*p* < 0.05) (**a**); the color scale shows the inhibition rate (%) at the given inhibitory concentration. The black color of the symbols indicates that inhibition rate values were not calculated. Non-toxic concentrations of fullerenes, NDs, GO, CNTs, and CDs (**b**); the percentage shown represents the proportion of non-toxic values relative to the total number of concentration values analyzed for each nanomaterial type.

Figure 10a presents the inhibitory concentration values of the five types of carbon nanomaterials for which the largest number of publications on antimicrobial activity were found (Figure 3a). Dunn’s post hoc test identified a statistically significant difference (*p* < 0.05) only between the NDs and CNTs samples. The interquartile ranges are 0.03–0.25 mg/mL for fullerenes, 0.016–50 mg/mL for NDs, 0.025–0.5 mg/mL for GO, 0.016–0.1 mg/mL for CNTs, and 0.03–0.27 mg/mL for CDs. Moskvitina et al. estimated the most commonly used range of carbon nanomaterial concentrations for antimicrobial activity analysis to be 50–500 μg/mL [84]. In our analysis, all carbon nanomaterial types except NDs exhibit typical inhibitory concentration ranges within the same order of magnitude. This consistency supports the representativeness of the collected data, despite the wide scatter of values (0.0005–1336 mg/mL for fullerenes; 0.004–500 mg/mL for NDs; 0.00025–29 mg/mL for GO; 0.0001–1 mg/mL for CNTs; 0.002–200 mg/mL for CDs). The median values are 0.1 mg/mL for fullerenes, CNTs, and CDs, 0.09 mg/mL for GO, and 0.25 mg/mL for NDs.

Evidence of non-toxicity toward bacteria and fungi exists for all types of carbon nanomaterials. In some cases, this refers to the pristine form compared to the functionalized form [67] or to combined application with other antimicrobial agents [83,109,110,179]; for fullerenes [65,133] and CDs [165], it refers, in some cases, to activity in the dark compared to photodynamic action. Figure 10b shows the concentration values that did not exhibit antimicrobial activity for the five carbon nanomaterial types. Non-toxic concentration values are determined less by the intrinsic properties of the nanomaterial and more by the specific concentration range selected by the researchers in a given study, making cross-type toxicity comparisons difficult based on these values. However, based on the proportion of non-toxicity cases relative to the total analyzed in this review, the absence of antimicrobial activity is most frequently observed for fullerenes (31%), followed by GO and NDs (approximately 15%), and then CNTs and CDs (approximately 10%).

It should be noted that carbon nanomaterials are capable of exhibiting a hormetic effect, stimulating bacterial growth at low concentrations (0.1–1 mg/mL) while inhibiting it at high concentrations (>10 mg/mL), as shown for GO against *E. coli* [180]. Furthermore, as shown for NDs against S. aureus, carbon nanomaterials can stimulate bacterial growth, even at a relatively high concentration of 10 mg/mL [180].

Overall, among the carbon nanomaterials reviewed, NDs exhibit the lowest antimicrobial activity, based on their interquartile range (Figure 10a) and the relatively few publications on this topic (Figure 1). Fullerenes, despite a higher number of reported non-toxicity cases (Figure 10b), are of interest for aPDT, particularly in their cationic form [36] (Figure 5). Their low dark toxicity may even enhance their potential as photosensitizers, as it increases the temporal and spatial selectivity of their action [65,181]. For the other three carbon nanomaterials—GO, CNTs, and CDs—antimicrobial activity is quite similar, both in terms of inhibitory concentration values (Figure 10a) and the proportion of non-toxicity cases (Figure 10b). The rapid recent growth in publications on CDs (Figure 1) is largely due to the wide variety of possible precursors and synthesis methods (Figure 2), which influence their structure, functional group composition, and antimicrobial properties. Furthermore, CDs offer advantages over GO and CNTs as antimicrobial agents, including somewhat lower toxicity to plants, animals, and humans (see Section 4.3) and greater controllability of their action in aPDT, similar to fullerenes [65].

When compared to other antimicrobial agents, the typical values of inhibitory concentrations of carbon nanomaterials (from tens to hundreds of μg/mL) are roughly comparable to those of ZnO nanoparticles against *E. coli* [47] and CuO nanoparticles against different bacteria [50], but are somewhat higher (i.e., less potent) than the typical values (up to 100 μg/mL) for ZnO against *S. aureus* [47], as well as for TiO_2_ nanoparticles [46], selenium [49], and Ag_2_O nanoparticles [48]. Silver nanoparticles generally show stronger antimicrobial activity than carbon nanomaterials [182,183]. Su et al. reported similar *E. coli* CFU reduction for MWNTs and silver nanoparticles, but at concentrations of 100 and 2 μg/mL, respectively [112]. Carbon nanomaterials appear even less effective when compared to conventional antibiotics and fungicides. For example, water-soluble fullerene derivatives have lower MICs than tetracyclines, even against tetracycline-resistant *E. coli*, but against a sensitive strain, their MICs are orders of magnitude higher [106]. A similar pattern—in which the antimicrobial activity of carbon nanomaterials is virtually negligible compared to antimicrobial agents that have specific molecular targets—has been demonstrated for the pairs NDs/vancomycin [184], GO/cyproconazole and GO/difenoconazole [179], CNTs/nisin [98], and CDs/ciprofloxacin [185]. Overall, the antimicrobial activity of carbon nanomaterials is moderate, as characterized for CNTs [172], especially pristine CNTs [146].

## 4. Mechanisms and Selectivity of Toxic Effects of Carbon Nanomaterials

### 4.1. Mechanisms of Toxicity of Carbon Nanomaterials

**Figure 11:**
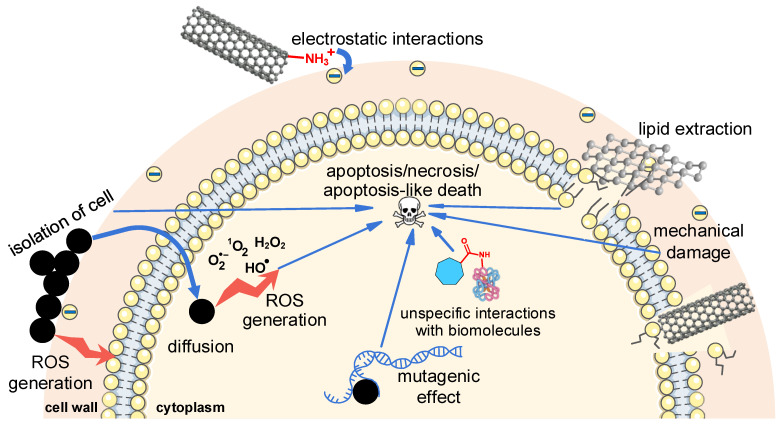
Generalized scheme of antimicrobial action mechanisms of carbon nanomaterials.

The primary toxicity mechanisms of carbon nanomaterials are illustrated in Figure 11. Direct contact between nanomaterials and cells is a key factor in antimicrobial activity [100,186]. For fullerenes, NDs, CNTs, and CDs, the strategy of enhancing cell contact by imparting a positive surface charge—primarily via amino group functionalization—is discussed above. Electrostatic attraction of positively charged particles to microbial cells arises because microbial cell walls typically carry a negative charge [65] (Figure 11, top); the same holds for bacterial flagella [54]. Moreover, the carbon framework, particularly the sp^2^-bonded hexagonal structure, is non-polar and poorly water-soluble [67,187,188]. Therefore, a major goal of functionalizing CNTs [189], fullerenes [106], and other carbon nanomaterials [188] is to enhance the stability of their aqueous suspensions by introducing hydrophilic polar groups, including negatively charged carboxyl groups. The presence of polar groups also largely explains why GO exhibits greater antimicrobial activity than less-oxidized reduced GO and graphene [68,100]. Polar groups shield highly hydrophobic surfaces, promote electrostatic repulsion between particles, reduce aggregation, improve dispersion [188], and increase the probability of collision between particles and cells in suspension [100,104]. Hydrogen bonding between nanomaterial functional groups and, for instance, hydroxyl groups of fungal cell wall glycoproteins may also facilitate nanomaterial attachment to cells [190]. Wang et al. proposed that van der Waals forces between CNTs could promote the formation of spore–CNT aggregates [100]. Interaction of particles with cells may also occur on a mechanical basis, for example, when shaking a co-suspension [54,104].

The formation of a coating around the entire cell by aggregated particles (e.g., CDs [99], NDs [54], and CNTs [100]; Figure 11, left) or by large 2D nanomaterial sheets (e.g., large-sized GO [54,104]) can cause a wrapping effect. This isolates the cell from its environment, leading to nutrient deprivation [99] and impaired cell division [158]. In the case of fungal spores, inhibition of germination may be related to disruption of water uptake [100,103]. Besides the wrapping effect, extensive adhesion of nanomaterials to cell walls promotes the clustering of cells into larger aggregates. Norouzi et al. demonstrated that this can reduce bacterial colony-forming ability while the turbidity of the suspension remains unchanged [95].

Upon contact with the cell wall, carbon nanomaterials can compromise their integrity through various mechanisms. Computer simulations with fullerenes indicate that, owing to their lipophilicity, carbon nanomaterials can penetrate the membrane bilayer, disrupt lipid packing, and form pores, thereby disturbing ion homeostasis [191,192]. Small-sized GO causes mechanical disruption via its sharp edges with dangling sp^3^-hybridized bonds; medium-sized sheets are capable of this only at specific favorable orientations, whereas large-sized sheets tend to adhere to and wrap around the lipid bilayer [104]. CNTs physically puncture cell walls (Figure 11, right), and this mechanism is associated with an increase in their toxicity as their length and diameter decrease [104,147]. While mechanical disruption is typical for GO and CNTs, it has also been demonstrated for large fullerene aggregates [191] and other carbon nanomaterials [104]. Furthermore, a distinct mechanism of cell membrane damage has been proposed for GO — the lipid extraction effect (Figure 11, top right), in which GO contacts the membrane not with its edge but with its basal plane [104]. GO sheets bind to lipid headgroups via electrostatic and other interactions, retaining sufficient rotational freedom to form hydrogen bonds with the C=O group in the phosphatide hydrophobic region. This allows for deeper insertion and capture of lipid alkyl chains through hydrophobic interactions; subsequent rotation extracts a large fraction of phospholipids onto the GO surface [193]. Carbon nanomaterials can also exhibit antimicrobial activity without cell wall rupture. Jira et al., for instance, found that disruptions play no significant role in the antibacterial activity of NDs and GO, as shown by electron microscopy and by the lack of enhanced activity on saline agar [54]. If significant cell wall ruptures occurred, increased salinity would intensify osmotic stress and raise the number of dead cells.

A common toxicity mechanism of carbon nanomaterials—observed for fullerenes [65,70], GO [142], CNTs [194], and CDs [167]—is ROS generation (Figure 11, left). Light-independent ROS formation arises from the oxidation of reducing agents such as NADH (β-nicotinamide adenine dinucleotide, reduced form) by carbon nanomaterials, with subsequent electron transfer to molecular oxygen to form the superoxide anion radical O_2_^•−^ [194]. The superoxide anion radical itself is a weak oxidant, but it can be protonated to the stronger perhydroxyl radical HO_2_^•^, which can then react with another O_2_^•−^ to produce hydrogen peroxide H_2_O_2_ [195] or with double bonds in phospholipid fatty acid residues, initiating lipid peroxidation [196]. In the presence of iron ions, the Fenton reaction converts H_2_O_2_ into the highly reactive hydroxyl radical HO^•^, capable of reacting with any biomolecule and triggering radical chain reactions [195]. In light-dependent ROS formation, the carbon nanomaterial acts as a photosensitizer: a valence electron is excited to the short-lived singlet state S_1_, which can undergo intersystem crossing to the longer-lived triplet state T_1_, which is sufficiently long-lived to oxidize a biomolecule with a suitable redox potential and subsequently form O_2_^•−^ (Type I mechanism of photodynamic action) or transfer energy to molecular oxygen, generating the strong oxidant singlet oxygen ^1^O_2_ (Type II mechanism) [104]. The efficiency of photodynamic action depends mainly on the singlet oxygen quantum yield [65], since ^1^O_2_, unlike O_2_^•−^ and H_2_O_2_ [197], is not neutralized by specific cellular enzymes and is quenched only by non-enzymatic antioxidants [198], while also requiring no additional steps to oxidize biomolecules. Exceeding a critical ROS threshold leads to oxidative distress in the cell [199]. Biomolecule oxidation can impair essential cellular functions (e.g., loss of plasma membrane integrity leading to necrosis) or, via signaling pathways, trigger programmed cell death — specifically, apoptosis in fungi cells [200] and apoptosis-like death in bacteria cells [201].

Oxidative damage may arise not from ROS generation by carbon nanomaterials, but from unspecific direct oxidation of biomolecules [104] (Figure 11, center). For instance, Brunet et al. suggested that membrane proteins are oxidized directly in aqueous fullerene suspensions prepared with tetrahydrofuran as a transitional solvent, as toxicity did not correlate with ROS generation efficiency [121]. Wehling et al. proposed that the reactivity of carboxylic acid groups in acid anhydride forms on the surface of detonation NDs is a key determinant of their antibacterial activity [72].

Carbon nanomaterials can also penetrate microbial cells and exert cytotoxicity from within, as shown for CDs [99,186] and NDs [72]. Deoxyribonucleic acid (DNA) damage has been reported for CDs [99] and CNTs [146], and fullerene-generated ^1^O_2_ has been shown to cause mutagenic effects [202] (Figure 11, bottom). DNA damage can also trigger apoptosis-like death [201].

Overall, the mechanisms of antimicrobial activity of carbon nanomaterials are characterized by low specificity of action, which reduces the likelihood of microorganisms developing resistance against them [12,147] and broadens their spectrum of action compared to conventional antibiotics that target specific sites [147]. Additionally, carbon nanomaterials can act through multiple pathways simultaneously, as demonstrated for GO through mechanical damage to the cell wall, insertion, and generation of intracellular ROS [142].

### 4.2. Selectivity of Antimicrobial Activity of Carbon Nanomaterials

“Microbes” is a collective term for a wide variety of microorganisms. In this review, it refers mainly to bacteria and fungi, but can also include archaea, protozoa, metazoans (primarily helminths), and viruses [203]. Prokaryotic bacterial cells are generally smaller than eukaryotic fungal cells, lack a nucleus and mitochondria, and differ in many other respects [204]. Studies of antimicrobial activity place special emphasis on differences in cell wall structure, as the cell wall is the primary barrier protecting the cell [205]. Gr− bacteria possess a cell wall composed of two phospholipid membranes with a relatively thin peptidoglycan layer sandwiched between them. Lipopolysaccharides are also present on the outer membrane surface [93,205]. Gr+ bacteria have only an inner membrane but a thicker peptidoglycan layer than Gr− bacteria; this layer contains numerous negative charges from the phosphate groups of teichoic acids, which may enhance the interaction of the cell wall with positively charged particles compared to Gr− bacteria [155,206]. Various polysaccharides and/or polypeptides are associated with the peptidoglycan layer [205]. The cell wall of fungi from the subkingdom Dikarya—the main representatives studied in this review (genera *Aspergillus*, *Candida*, *Saccharomyces*, and others)—consists of chitin and linear glucans near the cytoplasmic membrane and glucans and other polysaccharides farther from the membrane. These polysaccharides can be components of glycoproteins, and their composition may vary among different genera [186,205].

**Figure 12:**
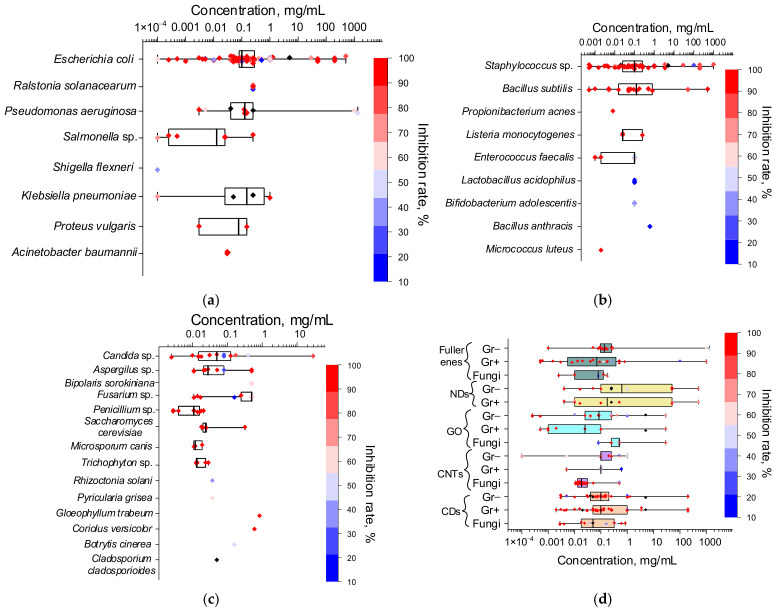
Inhibitory concentration values of carbon nanomaterials against different species of Gr− bacteria (**a**), Gr+ bacteria (**b**), and fungi (**c**). Inhibitory concentration values of fullerenes, NDs, GO, CNTs, and CDs against specific microbial groups, Gr− bacteria, Gr+ bacteria, and fungi (**d**). The color scale shows the inhibition rate (%) at the given inhibitory concentration. The black color of the symbols indicates that inhibition rate values were not calculated.

For carbon nanomaterials, the selectivity of their antimicrobial action has been demonstrated in some studies: for example, milled NDs exhibit higher antimicrobial activity against Gr+ bacteria than against Gr− bacteria in [93]; physical punctures of SWNTs also have a more pronounced effect against Gr+ bacteria than against Gr− with a stiffer cell wall in [144]; and CDs are more toxic against oomycetes compared to fungi in [6]. Nevertheless, in most studies, no selective toxicity of carbon nanomaterials toward specific groups or genera of microorganisms has been found. Analysis of the collected data also did not reveal such selectivity of carbon nanomaterials with respect to genera within groups of microorganisms (Gr− bacteria (Figure 12a), Gr+ bacteria (Figure 12b), and fungi/fungi-like microorganisms (Figure 12c)). No statistically significant difference in antimicrobial activity against whole groups of microbes was detected when comparing fullerenes, NDs, GO, CNTs, and CDs separately (Figure 12d). It should only be noted that, although there is evidence that NDs reduce the adhesion of *C. albicans* to polymethyl methacrylate nanocomposites [207,208], no studies have been found that demonstrate their antifungal activity in suspension, which may indirectly indicate their lower toxicity toward fungi than toward bacteria.

Based on the analyzed data, no significant differences were found in the influence of the physicochemical properties (size and zeta-potential) of carbon nanomaterials on antimicrobial activity against different groups of microorganisms. The observed trends for CNTs (Figure 8b) and CDs (Figure 9b) are approximately similar regarding all groups of microorganisms.

### 4.3. Toxicity to Plants, Animals and Humans

The low specificity of the toxic mechanisms of carbon nanomaterials has a downside: potential hazard to plants and animals, especially because these materials are stable enough to accumulate in the environment [209]. Cytotoxicity to humans is particularly critical when considering them as nanoantibiotics for medical use [17]. However, unlike purely disinfecting agents such as hypochlorites [210] or hydrogen peroxide [211], nanomaterials are expected to cause not acute toxicity at their working concentrations, but rather delayed effects upon long-term exposure [17]. Carbon nanomaterials are able to exhibit toxicity toward mammalian cells in vitro in roughly the same range (0.1–1 mg/mL) as toward bacterial or fungal cultures [212]. CNTs most frequently show toxicity toward various mammalian cell types [213], as well as toward plants and animals [214]. Functionalized CNTs may be less toxic than pristine ones [101,144], but toxicity is still observed, sometimes even higher than that of pristine CNTs [215]. GO has been reported to be toxic to reproductive cells [216], neurotoxic (crossing the blood–brain barrier), as well as hepatotoxic, nephrotoxic, and dermatotoxic [213]. NDs exhibit lower toxicity; detonation NDs are biocompatible with various mammalian cell types [217,218], although genotoxicity [209], cardiotoxicity [213], and macrophage toxicity [219] have also been reported. Fullerenes are generally considered of low toxicity in the ground state [65] and can even show antioxidant properties, e.g., reducing anthracycline-induced cardiotoxicity [220] and genetic abnormalities in ovarian cells [221]; however, hepatotoxicity [213] and toxicity to vascular endothelial cells have also been found [222]. The toxicity of CDs to mammalian cells varies greatly due to their diversity. In the studies analyzed here, most often either no toxicity was detected [6,61,99,157], or cell viability was at least 80% at concentrations where antimicrobial activity was observed [74,115,154,156,158,159,161]. While some CDs show toxicity to fibroblasts—for instance, citric acid-derived CDs reduced fibroblast viability to 35% in vitro [160] —others, such as chlorhexidine-gluconate-derived CDs, were found to be non-toxic [155]. The in vitro toxicity has also been reported against human cancer cells [120] and epithelial cells [212]. Upon photoexcitation, the toxicity of CDs [74], like that of fullerenes [65], increases markedly toward cell cultures; however, antimicrobial photodynamic therapy is mainly a localized, time-controlled process, which reduces undesirable cytotoxicity in vivo [65]. The toxicity of carbon nanomaterials to plants, animals, and humans is discussed in more detail elsewhere [209,212,213,214,216,223].

It is worth noting that the potential use of carbon nanomaterials (GO, CNTs, CDs, and others) as plant biostimulants, whether to enhance photosynthesis and stress tolerance [224] or to increase the abundance of plant-beneficial microorganisms in the soil [225], is currently being actively explored.

### 4.4. Prospects of Carbon Nanomaterials as Antimicrobial Agents

Numerous strategies exist that modify the properties of carbon nanomaterials more extensively than those discussed in this review (Figure 2). These strategies aim to increase toxicity against undesirable microorganisms while reducing toxicity toward host cells (in infection treatment [226]) and toward plants, animals, and humans more broadly. For instance, carbon nanomaterials can be used simultaneously with other antimicrobial agents, potentially achieving synergy in part because the nanomaterials can act as carriers [227]. Examples include silver nanoparticles combined with fullerenes [228], NDs [229], GO [109], CNTs [110,112], or CDs [182], carbon nanomaterials combined with black phosphorus nanosheets [126], with nanoparticles of metals other than Ag and their oxides [83,230], with conventional antiseptics [231], with organic antibiotics [98,232,233], and with fungicides [74,179]. In aPDT, fullerene conjugates with antenna molecules have been developed for excitation in the red and near-infrared ranges, which are less absorbed by biological tissues [36]. Enhanced toxicity can also be achieved by irradiating GO with ultraviolet light [78] or CNTs with microwaves [234]; photothermal [74] and photocatalytic effects [235], and even electric current [236], can be employed. Antimicrobial activity can also be increased by enhancing the affinity of carbon nanomaterials for microbial cells through active targeting, e.g., by conjugation with glucans [237]. Modifications may also aim to reduce toxicity to mammalian cells; for instance, polyacrylic acid-modified GO was found to be less toxic than the pristine form [238].

In biomedicine, carbon nanomaterials can be effective against other microbes not covered here, such as viruses [226], they can penetrate biofilms and inhibit their formation [186,237], they can capture pathogens [239], and they are being explored as components of implant composites [116,240] due to their antifouling properties [241]. Even if the insufficiently selective toxicity of CNTs and GO limits their use for treating infections, their antimicrobial properties remain valuable for food packaging composites [82], antifouling materials for various applications [242], water purification filters [98,243,244,245], water disinfection [236], and as fungicides in agriculture [179,246]. Moreover, in biomedicine, carbon nanomaterials can serve not as antimicrobial agents, but as drug carriers, immunoadjuvants, detection probes, tissue engineering vectors, biosensor components, and agents for cancer treatment [53,247,248,249,250].

**Table 1:** Data on the physico-chemical properties and antimicrobial activity of various carbon nanomaterials.

Line Number	Type of Carbon Nanomaterial	Details About Nanomaterial ^1^ and Experiment	Size ^2^, nm	Zeta-Potential, mV	Microorganism		Antimicrobial Effect	Concentration, mg/mL	Inhibition Rate, %	Microbiological Parameter	Reference
					Group	Species					
1	Fullerenes	fulleropyrrolidine, aPDT			Gr−	*Escherichia coli*	no	0.001		CFU count	[133]
2					Gr+	*Staphylococcus aureus*	yes	0.0005	92		
3					Fungi	*Candida albicans*	no	0.0025			
4		fulleropyrrolidine, cationic groups, aPDT			Gr−	*Escherichia coli*	yes	0.001	99		
5					Gr+	*Staphylococcus aureus*	yes	0.0005	99		
6					Fungi	*Candida albicans*	yes	0.0025	99		
7	Fullerenes	encapsulation in polymeric nanoparticles	66		Gr−	*Escherichia coli*	yes	0.25	100	CFU count	[29]
8					Gr+	*Bacillus subtilis*	yes	0.125	64.4		
9					Gr+	*Staphylococcus aureus*	yes	0.25	35.8		
10					Fungi	*Candida* sp.	yes	0.125	90.5		
11		encapsulation in polymeric nanoparticles with NDs	102		Gr−	*Escherichia coli*	yes	0.175	93.3		
12					Gr+	*Bacillus subtilis*	yes	0.175	93.3		
13					Gr+	*Staphylococcus aureus*	yes	0.175	100		
14					Fungi	*Candida* sp.	yes	0.175	83.4		
15	Fullerenes	encapsulation in polymeric nanoparticles			Gr−	*Escherichia coli*	yes	0.1	80	CFU count	[121]
16		encapsulation in polymeric nanoparticles, aPDT					yes	0.1	90		
17		hydroxylation	122				no	0.16			
18		hydroxylation, aPDT	122				no	0.16			
19		stirring in water	84				no	0.1			
20		stirring in water, aPDT	84				no	0.1			
21		tetrahydrofuran as the intermediate solvent	64				yes	0.1	95		
22		tetrahydrofuran as the intermediate solvent, aPDT	64				yes	0.1	100		
23	Fullerenes	pentasubstituted, anionic groups	70	−52.2	Gr−	*Escherichia coli*	no	0.57		CFU count	[70]
24		tetrasubstituted, cationic groups	11	41.5			yes	0.28	77.6		
25	Fullerenes	chitosan-coated glycine-fullerene conjugate	300	−25.2	Gr+	*Propionibacterium acnes*	yes	0.008	100	OD	[73]
26	Fullerenes	fulleropyrrolidine, cationic groups			Gr−	*Pseudomonas aeruginosa*	no	0.12		CFU count	[65]
27		fulleropyrrolidine, cationic groups, aPDT					yes	0.12	90		
28		fulleropyrrolidine, cationic groups, aPDT			Gr−	*Escherichia coli*	yes	0.08	99		
29		fulleropyrrolidine, cationic groups					no	0.12			
30		fulleropyrrolidine, cationic groups, aPDT			Gr+	*Staphylococcus aureus*	yes	0.0006	99		
31		fulleropyrrolidine, cationic groups					yes	0.013	99		
32		fulleropyrrolidine, cationic groups, aPDT			Fungi	*Candida albicans*	yes	0.01	99		
33		fulleropyrrolidine, cationic groups					no	0.12			
34	Fullerenes	hydroxylation	36.1 ^3^	−13.9 ^3^	Gr−	*Pseudomonas aeruginosa*	yes	1000	60	CFU count	[124]
35					Gr+	*Staphylococcus aureus*	yes	1000	82		
36		stirring in water	190 ^3^	−38 ^3^	Gr−	*Pseudomonas aeruginosa*	yes	1336	50		
37					Gr+	*Staphylococcus aureus*	yes	100	27		
38	Fullerenes	encapsulation in polymeric nanoparticles	25		Gr+	*Bacillus subtilis*	yes	0.8	100	OD	[119]
39		stirring in water	74.9				yes	0.5	100		
40		tetrahydrofuran as the intermediate solvent	75.6				yes	0.09	100		
41		toluene as the intermediate solvent	25				yes	0.5	100		
42	Fullerenes	fullerene C_70_ cyclopropane, cationic groups			Gr−	*Escherichia coli*	yes	0.25	99	CFU count	[89]
43			80		Gr+	*Staphylococcus aureus*	no	0.03			
44		fullerene C_70_ cyclopropane, cationic groups, aPDT			Gr−	*Escherichia coli*	yes	0.09	99		
45			80		Gr+	*Staphylococcus aureus*	yes	0.003	99		
46		fullerene cyclopropane, cationic groups			Gr−	*Escherichia coli*	yes	0.27	99		
47			80		Gr+	*Staphylococcus aureus*	no	0.03			
48		fullerene cyclopropane, cationic groups, aPDT			Gr−	*Escherichia coli*	yes	0.15	99		
49			80		Gr+	*Staphylococcus aureus*	yes	0.0015	99		
50	Fullerenes	stirring in water			Gr−	*Ralstonia solanacearum*	no	0.25		OD	[55]
51	Fullerenes	stirring in water			Fungi	*Fusarium graminearum*	no	0.5		Mycological	[100]
52					Fungi	*Fusarium poae*	no	0.5			
53	Fullerenes	encapsulation of C_70_ in polymeric nanoparticles			Gr-	*Escherichia coli*	no	0.34		CFU count	[67]
54					Gr+	*Staphylococcus aureus*	no	0.34			
55		hydroxylation of C_70_			Gr−	*Escherichia coli*	no	0.46			
56					Gr+	*Staphylococcus aureus*	no	0.46			
57		octasubstituted C_70_, cationic groups	116.2	44.7	Gr−	*Escherichia coli*	yes	0.05	90		
58			116.2	44.7	Gr+	*Staphylococcus aureus*	yes	0.02	90		
59		pentasubstituted C_70_, cationic groups			Gr−	*Escherichia coli*	yes	0.15	90		
60					Gr+	*Staphylococcus aureus*	yes	0.05	90		
61		three-substituted C_70_, cationic groups			Gr−	*Escherichia coli*	yes	0.09	90		
62					Gr+	*Staphylococcus aureus*	yes	0.04	90		
63	Fullerenes	pentasubstituted, anionic groups	65		Gr−	*Escherichia coli*	no	5 ^5^		OD	[106]
64		pentasubstituted, cationic groups	91	20.5 ^4^			yes	0.261	100		
65	Fullerenes	hydroxylation	20	−7	Fungi	*Aspergillus flavus*	yes	0.08	27	Mycological	[71]
66	Nanodiamonds	detonation, amination	78	35.3	Gr+	*Staphylococcus aureus*	yes	0.01	72	OD	[136]
67		detonation, carboxylation	90	−35.5			no	0.01			
68		detonation, hydroxylation	89	22.60			no	0.01			
69	Nanodiamonds	detonation	5		Gr−	*Escherichia coli*	yes	0.05	100	CFU count	[251]
70	Nanodiamonds	Hummer’s method and cavitation			Gr−	*Escherichia coli*	yes	0.25		ZOI	[97]
71					Gr−	*Klebsiella pneumoniae*	yes	0.25			
72					Gr−	*Pseudomonas aeruginosa*	yes	0.25			
73					Gr+	*Staphylococcus aureus*	yes	0.25			
74	Nanodiamonds	detonation, annealing, experiment in LB medium ^6^	5	−15	Gr−	*Escherichia coli*	yes	1	35	CFU count	[54]
75		detonation, annealing, experiment in MH medium ^6^	5	−10			no	1			
76		detonation, experiment in LB medium ^6^	5	−7			yes	1	45		
77		detonation, experiment in MH medium ^6^	5	−18			yes	1	55		
78	Nanodiamonds	detonation	5		Gr−	*Escherichia coli*	yes	0.1	100	CFU count	[84]
79					Gr+	*Staphylococcus epidermidis*	yes	0.1	100		
80	Nanodiamonds	milled	138		Gr−	*Escherichia coli*	no	0.5		CFU count	[95]
81					Gr+	*Staphylococcus aureus*	yes	0.5	98		
82					Gr+	*Staphylococcus epidermidis*	yes	0.01	95		
83	Nanodiamonds	milled	138		Gr−	*Escherichia coli*	no	0.5		CFU count	[93]
84					Gr+	*Staphylococcus aureus*	yes	0.01	70		
85	Nanodiamonds	detonation, carboxylation	4.2		Gr−	*Escherichia coli*	yes	0.004	40	OD	[90]
86					Gr−	*Escherichia coli*	yes	0.016	99.9	CFU count	
87					Gr+	*Streptococcus mutans*	yes	0.016	99.9	CFU count	
88					Gr+	*Streptococcus mutans*	yes	0.004	30	OD	
89	Nanodiamonds	detonation	4		Gr−	*Escherichia coli*	yes	50	92	CFU count	[72]
90					Gr+	*Bacillus subtilis*	yes	500	90		
91		detonation, annealing	4	−44	Gr−	*Escherichia coli*	yes	50	95		
92					Gr+	*Bacillus subtilis*	yes	50	90		
93		detonation, annealing in H_2_	4	63	Gr−	*Escherichia coli*	yes	500	80		
94					Gr+	*Bacillus subtilis*	yes	500	70		
95		detonation, ultrasound	4		Gr−	*Escherichia coli*	yes	50	95		
96					Gr+	*Bacillus subtilis*	yes	50	88		
97	Graphene oxide	graphite oxidation and exfoliation			Fungi	*Candida albicans*	yes	0.08	23	OD	[111]
98					Fungi	*Candida tropical*	yes	0.08	22		
99	Graphene oxide	bio-reduction of GO		−21.2	Gr−	*Escherichia coli*	yes	0.04	96.6	CFU count	[139]
100		graphite oxidation and exfoliation		−31			yes	0.04	80.39		
101	Graphene oxide	graphite oxidation and exfoliation			Gr−	*Escherichia coli*	no	0.05		ZOI	[110]
102					Gr+	*Staphylococcus aureus*	no	0.05			
103	Graphene oxide	graphite oxidation and exfoliation			Gr−	*Escherichia coli*	yes	29	75	CFU count	[252]
104					Gr+	*Staphylococcus aureus*	yes	29	88		
105					Fungi	*Candida albicans*	yes	29	80		
106	Graphene oxide	graphite oxidation and exfoliation			Gr−	*Escherichia coli*	yes	0.085	98.5	CFU count	[253]
107		reduction of GO					yes	0.085	90		
108	Graphene oxide	graphite oxidation and exfoliation, experiment in LB medium ^6^	1250 ^7^	−28	Gr−	*Escherichia coli*	yes	1	40	CFU count	[54]
109		graphite oxidation and exfoliation, experiment in MH medium ^6^	1250 ^7^	−31	Gr−	*Escherichia coli*	no	1			
110		reduction of GO, experiment in LB medium ^6^	550 ^7^	−22	Gr−	*Escherichia coli*	yes	1	30		
111		reduction of GO, experiment in MH medium ^6^	550 ^7^		Gr−	*Escherichia coli*	no	1			
112	Graphene oxide	graphite oxidation and exfoliation	850 ^8^	−49.8	Gr−	*Salmonella enterica*	yes	0.025	100	CFU count	[68]
113					Gr+	*Listeria monocytogenes*	yes	0.025	100	CFU count	
114		reduction of GO	650 ^8^	−25.1	Gr−	*Salmonella enterica*	yes	0.25	100	CFU count	
115					Gr+	*Listeria monocytogenes*	yes	0.025	100	CFU count	
116	Graphene oxide	graphite oxidation and exfoliation	310		Gr−	*Escherichia coli*	yes	0.08	90	CFU count	[62]
117		reduction of GO	2750				yes	0.08	77		
118	Graphene oxide	reduction of GO	1500 ^7^		Gr−	*Escherichia coli*	yes	0.4	70	CFU count	[138]
119	Graphene oxide	reduction of GO			Gr−	*Escherichia coli*	no	5		ZOI	[82]
120					Gr+	*Staphylococcus aureus*	no	5			
121	Graphene oxide	graphite oxidation and exfoliation	90		Gr+	*Staphylococcus aureus*	yes	0.1	93	CFU count	[142]
122	Graphene oxide	reduction of GO			Fungi	*Aspergillus oryzae*	yes	0.5	100	Mycological	[190]
123					Fungi	*Aspergillus* *niger*	yes	0.5	100		
124					Fungi	*Fusarium oxysporum*	yes	0.25	100		
125	Graphene oxide	graphite oxidation and exfoliation			Gr−	*Escherichia coli*	yes	0.00025	100	OD	[78]
126							yes	0.0005	100	CFU count	
127					Gr−	*Salmonella typhimurium*	yes	0.00025	100	OD	
128							yes	0.0005	100	CFU count	
129					Gr+	*Bacillus subtilis*	yes	0.0005	100	OD	
130							yes	0.001	100	CFU count	
131					Gr+	*Enterococcus faecalis*	yes	0.001	100	OD	
132							yes	0.002	100	CFU count	
133	Graphene oxide	reduction of GO			Gr−	*Ralstonia solanacearum*	yes	0.25	10	OD	[55]
134							yes	0.25	25	CFU count	
135		graphite oxidation and exfoliation					yes	0.25	60	OD	
136							yes	0.25	85	CFU count	
137	Graphene oxide	graphite oxidation and exfoliation			Fungi	*Fusarium graminearum*	yes	0.5	60	Mycological	[100]
138					Fungi	*Fusarium poae*	yes	0.5	80		
139		reduction of GO			Fungi	*Fusarium graminearum*	yes	0.5	35		
140					Fungi	*Fusarium poae*	yes	0.5	55		
141	Graphene oxide	graphite oxidation and exfoliation			Fungi	*Fusarium graminearum*	no	0.005		Mycological	[179]
142	Graphene oxide	graphite oxidation and exfoliation			Gr−	*Escherichia coli*	yes	0.01	38	CFU count	[109]
143	Graphene oxide	graphite oxidation and exfoliation			Fungi	*Bipolaris sorokiniana*	yes	0.5	55	Mycological	[103]
144	Carbon nanotubes	MWNTs	3000		Gr−	*Escherichia coli*	no	2		CFU count	[234]
145		MWNTs, conjugation with 1-octadecanol groups	3000				no	2			
146	Carbon nanotubes	MWNTs			Gr+	*Staphylococcus aureus*	no	0.5		CFU count	[254]
147	Carbon nanotubes	MWNTs, carboxylation	10,000		Gr−	*Escherichia coli*	no	4		CFU count	[86]
148			10,000		Gr+	*Staphylococcus aureus*	no	4			
149	Carbon nanotubes	MWNTs, carboxylation	1250		Gr−	*Escherichia coli*	yes	0.1	30	OD	[147]
150					Gr+	*Lactobacillus acidophilus*	yes	0.1	15		
151					Gr+	*Bifidobacterium adolescentis*	yes	0.1	42		
152					Gr+	*Enterococcus faecalis*	yes	0.1	50		
153					Gr+	*Staphylococcus aureus*	yes	0.1	60		
154		MWNTs, hydroxylation	1250		Gr−	*Escherichia coli*	yes	0.1	35		
155					Gr+	*Lactobacillus acidophilus*	yes	0.1	15		
156					Gr+	*Bifidobacterium adolescentis*	yes	0.1	50		
157					Gr+	*Enterococcus faecalis*	yes	0.1	60		
158					Gr+	*Staphylococcus aureus*	yes	0.1	60		
159		MWNTs, long length	50,000		Gr−	*Escherichia coli*	yes	0.1	25		
160					Gr+	*Lactobacillus acidophilus*	no	0.1			
161					Gr+	*Bifidobacterium adolescentis*	yes	0.1	20		
162					Gr+	*Enterococcus faecalis*	yes	0.1	35		
163					Gr+	*Staphylococcus aureus*	yes	0.1	40		
164		MWNTs, short length	1250		Gr−	*Escherichia coli*	yes	0.1	25		
165					Gr+	*Lactobacillus acidophilus*	yes	0.1	12		
166					Gr+	*Bifidobacterium adolescentis*	yes	0.1	20		
167					Gr+	*Enterococcus faecalis*	yes	0.1	50		
168					Gr+	*Staphylococcus aureus*	yes	0.1	60		
169		SWNTs	1500		Gr−	*Escherichia coli*	yes	0.1	45		
170					Gr+	*Lactobacillus acidophilus*	yes	0.1	25		
171					Gr+	*Bifidobacterium adolescentis*	yes	0.1	50		
172					Gr+	*Enterococcus faecalis*	yes	0.1	50		
173					Gr+	*Staphylococcus aureus*	yes	0.1	70		
174	Carbon nanotubes	MWNTs, carboxylation			Gr−	*Escherichia coli*	no	0.05		ZOI	[110]
175					Gr+	*Staphylococcus aureus*	no	0.05			
176	Carbon nanotubes	MWNTs	10,000		Gr+	*Bacillus anthracis*	yes	0.6	17.82	fluorescence intensity of propidium iodide and SYTO9	[98]
177							yes	0.6	18.91		
178	Carbon nanotubes	MWNTs	5000		Gr−	*Escherichia coli*	yes	0.2	99	CFU count	[255]
179	Carbon nanotubes	MWNTs from kaolin clay			Gr−	*Shigella flexneri*	yes	0.0001	40	CFU count	[256]
180					Gr−	*Escherichia coli*	yes	0.0001	60		
181					Gr−	*Klebsiella pneumoniae*	yes	0.0001	70		
182					Gr−	*Salmonella sp*	yes	0.0001	70		
183	Carbon nanotubes	SWNTs with Tween-20	1000		Gr−	*Escherichia coli*	yes	0.005	60	CFU count	[144]
184					Gr−	*Pseudomonas aeruginosa*	yes	0.005	66		
185					Gr+	*Bacillus subtilis*	yes	0.005	88		
186					Gr+	*Staphylococcus aureus*	yes	0.005	85		
187	Carbon nanotubes	MWNTs, oxidation			Gr−	*Escherichia coli*	yes	1	75	CFU count	[145]
188		SWNTs, oxidation					yes	1	50	CFU count	
189	Carbon nanotubes	MWNTs, long length, large diameter	1000		Gr−	*Escherichia coli*	yes	0.1	43	CFU count	[84]
190					Gr+	*Staphylococcus aureus*	yes	0.1	42		
191		MWNTs, long length, medium diameter	1000		Gr−	*Escherichia coli*	yes	0.1	56		
192					Gr+	*Staphylococcus aureus*	yes	0.1	40		
193		MWNTs, long length, small diameter	1000		Gr−	*Escherichia coli*	yes	0.1	52		
194					Gr+	*Staphylococcus aureus*	yes	0.1	56		
195		MWNTs, short length, medium diameter	500		Gr−	*Escherichia coli*	yes	0.1	57		
196					Gr+	*Staphylococcus aureus*	yes	0.1	52		
197		MWNTs, oxidation, short length, medium diameter	500		Gr−	*Escherichia coli*	yes	0.1	100		
198					Gr+	*Staphylococcus aureus*	yes	0.1	100		
199		MWNTs, short length, small diameter	600		Gr−	*Escherichia coli*	yes	0.1	68		
200					Gr+	*Staphylococcus aureus*	yes	0.1	52		
201		MWNTs, oxidation, short length, small diameter	600		Gr−	*Escherichia coli*	yes	0.1	98		
202					Gr+	*Staphylococcus aureus*	yes	0.1	99		
203	Carbon nanotubes	MWNTs, oxidation			Gr−	*Methylobacterium spp.*	no	1		CFU count	[102]
204					Gr−	*Sphingomonas spp.*	no	1			
205	Carbon nanotubes	MWNTs, oxidation	2000		Gr−	*Escherichia coli*	yes	0.1	82	CFU count	[112]
206	Carbon nanotubes	MWNTs	15,000		Gr−	*Escherichia coli*	no	0.512		CFU count	[83]
207							no	0.5		OD	
208		MWNTs, carboxylation	15,000				yes	0.512	68	CFU count	
209							yes	0.5	15	OD	
210	Carbon nanotubes	MWNTs			Gr−	*Ralstonia solanacearum*	yes	0.25	40	OD	[55]
211					Gr−	*Ralstonia solanacearum*	yes	0.25	59	CFU count	
212		SWNTs			Gr−	*Ralstonia solanacearum*	yes	0.25	75	OD	
213					Gr−	*Ralstonia solanacearum*	yes	0.25	91	CFU count	
214	Carbon nanotubes	MWNTs			Fungi	*Fusarium graminearum*	yes	0.5	49	Mycological	[100]
215					Fungi	*Fusarium poae*	yes	0.5	64		
216		SWNTs			Fungi	*Fusarium graminearum*	yes	0.5	64		
217					Fungi	*Fusarium poae*	yes	0.5	63		
218	Carbon nanotubes	MWNTs	50,000		Fungi	*Fusarium graminearum*	yes	0.5	33	Mycological	[101]
219		MWNTs, amination	50,000				yes	0.5	85		
220		MWNTs, carboxylation	50,000				yes	0.5	80		
221		MWNTs, hydroxylation	50,000				yes	0.5	90		
222	Carbon nanotubes	arginine-MWNTs conjugate	5000		Fungi	*Aspergillus fumigatus*	yes	0.011	100	OD	[146]
223					Fungi	*Aspergillus niger*	yes	0.023	100		
224					Fungi	*Candida albicans*	yes	0.015	100		
225					Fungi	*Fusarium culmorum*	yes	0.011	100		
226					Fungi	*Microsporum canis*	yes	0.011	100		
227					Fungi	*Penicillium chrysogenum*	yes	0.014	100		
228					Fungi	*Penicillium lilacinum*	yes	0.011	100		
229					Fungi	*Saccharomyces cerevisiae*	yes	0.018	100		
230					Fungi	*Trichophyton mentagrophytes*	yes	0.013	100		
231					Fungi	*Trichophyton rubrum*	yes	0.013	100		
232		lysine-MWNTs conjugate	5000		Fungi	*Aspergillus fumigatus*	yes	0.011	100		
233					Fungi	*Aspergillus niger*	yes	0.028	100		
234					Fungi	*Candida albicans*	yes	0.015	100		
235					Fungi	*Fusarium culmorum*	yes	0.014	100		
236					Fungi	*Microsporum canis*	yes	0.012	100		
237					Fungi	*Penicillium chrysogenum*	yes	0.016	100		
238					Fungi	*Penicillium lilacinum*	yes	0.011	100		
239					Fungi	*Saccharomyces cerevisiae*	yes	0.02	100		
240					Fungi	*Trichophyton mentagrophytes*	yes	0.014	100		
241					Fungi	*Trichophyton rubrum*	yes	0.013	100		
242		MWNTs	5000		Fungi	*Aspergillus fumigatus*	yes	0.021	100		
243					Fungi	*Aspergillus niger*	yes	0.051	100		
244					Fungi	*Candida albicans*	yes	0.031	100		
245					Fungi	*Fusarium culmorum*	yes	0.017	100		
246					Fungi	*Microsporum canis*	yes	0.019	100		
247					Fungi	*Penicillium chrysogenum*	yes	0.018	100		
248					Fungi	*Penicillium lilacinum*	yes	0.021	100		
249					Fungi	*Saccharomyces cerevisiae*	yes	0.025	100		
250					Fungi	*Trichophyton mentagrophytes*	yes	0.029	100		
251					Fungi	*Trichophyton rubrum*	yes	0.024	100		
252	Carbon dots	antimicrobial agent-derived, N-doped	8		Gr−	*Klebsiella* *pneumoniae*	yes	0.05		ZOI	[92]
253					Gr−	*Pseudomonas aeruginosa*	yes	0.04			
254					Gr+	*Staphylococcus aureus*	yes	0.02			
255	Carbon dots	antimicrobial agent-derived, temperature of synthesis 120 °C	4		Fungi	*Penicillium italicum*	yes	0.0028	100	CFU count	[85]
256							yes	0.0028	100	OD	
257		antimicrobial agent-derived, temperature of synthesis 180 °C	4				yes	0.004	100	CFU count	
258	Carbon dots	N-doped, aPDT	6	14	Gr−	*Escherichia coli*	yes	0.1	99	CFU count	[165]
259		N-doped					no	0.4			
260	Carbon dots	antimicrobial agent-derived, N-doped	14	9	Gr−	*Escherichia coli*	yes	0.05	92	CFU count	[61]
261							yes	0.003	100	OD	
262					Gr−	*Proteus vulgaris*	yes	0.003	100	OD	
263					Gr−	*Pseudomonas aeruginosa*	yes	0.003	100	OD	
264					Gr+	*Micrococcus luteus*	yes	0.002	100	OD	
265					Gr+	*Staphylococcus aureus*	yes	0.003	100	OD	
266							yes	0.05	93	CFU count	
267					Gr+	*Staphylococcus epidermidis*	yes	0.004	100	OD	
268					Fungi	*Candida albicans*	yes	0.018	100	OD	
269							yes	0.05	96	CFU count	
270					Fungi	*Saccharomyces cerevisiae*	yes	0.024	100	OD	
271	Carbon dots	top-down synthesis from CNTs			Fungi	*Alternaria alternata*	no	0.16		OD	[6]
272					Fungi	*Botrytis cinerea*	yes	0.16	48	OD	
273					Fungi	*Fusarium oxysporum*	yes	0.16	17	OD	
274					Fungi	*Phytophthora infestans*	yes	0.02	87	Mycological	
275							yes	0.16	70	OD	
276	Carbon dots	from vitamin C	4.5		Gr−	*Escherichia coli*	yes	0.075	100	CFU count	[99]
277							yes	0.075	100	OD	
278					Gr+	*Bacillus subtilis*	yes	0.05	100	CFU count	
279							yes	0.05	100	OD	
280					Gr+	*Staphylococcus aureus*	yes	0.1	100	CFU count	
281							yes	0.1	100	OD	
282					Fungi	*Pyricularia grisea*	yes	0.0375	64	Mycological	
283					Fungi	*Rhizoctonia solani*	yes	0.0375	33	Mycological	
284	Carbon dots	antimicrobial agent-derived, N-doped	1.27		Gr−	*Escherichia coli*	yes	0.064	90	CFU count	[156]
285							yes	0.064	100	OD	
286					Gr−	*Pseudomonas aeruginosa*	yes	0.128	80	CFU count	
287							yes	0.128	100	OD	
288					Gr+	*Bacillus subtilis*	yes	0.064	100	OD	
289							yes	0.128	99	CFU count	
290					Gr+	*Staphylococcus aureus*	yes	0.064	70	CFU count	
291							yes	0.128	100	OD	
292	Carbon dots	antimicrobial agent-derived, aPDT	3.31		Gr−	*Acinetobacter baumannii*	yes	0.03	99	CFU count	[157]
293		antimicrobial agent-derived				*Acinetobacter baumannii*	yes	0.03	96.7	CFU count	
294						*Acinetobacter baumannii*	yes	0.032	100	OD	
295		antimicrobial agent-derived, aPDT			Gr+	*Staphylococcus aureus*	yes	0.03	98	CFU count	
296		antimicrobial agent-derived					yes	0.03	92.5	CFU count	
297							yes	0.064	100	OD	
298	Carbon dots	from milk vetch leaves, N-doped	5	−20	Gr−	*Escherichia coli*	yes	1	30	OD	[158]
299					Gr+	*Staphylococcus aureus*	yes	1	30		
300		from osmanthus leaves, antimicrobial agent-derived, N-doped	6.50	−20	Gr−	*Escherichia coli*	yes	1	87		
301					Gr+	*Staphylococcus aureus*	yes	1	97		
302		from tea leaves, antimicrobial agent-derived, N-doped	5	−20	Gr−	*Escherichia coli*	yes	1	62		
303					Gr+	*Staphylococcus aureus*	yes	1	90		
304	Carbon dots	Cl-doped, N-doped	5		Gr−	*Escherichia coli*	yes	200	94	CFU count	[161]
305							no	0.031		OD	
306					Gr+	*Staphylococcus aureus*	yes	200	84	CFU count	
307							no	0.031		OD	
308		Cl-doped, N-doped, aPDT			Gr−	*Escherichia coli*	no	200		CFU count	
309					Gr+	*Staphylococcus aureus*	yes	200	84	CFU count	
310		F-doped, N-doped	5		Gr−	*Escherichia coli*	yes	200	94	CFU count	
311							no	0.031		OD	
312					Gr+	*Staphylococcus aureus*	yes	200	87	CFU count	
313							no	0.031		OD	
314		F-doped, N-doped, aPDT			Gr−	*Escherichia coli*	no	200		CFU count	
315					Gr+	*Staphylococcus aureus*	yes	200	99	CFU count	
316	Carbon dots	N-doped	3.1		Gr−	*Escherichia coli*	yes	0.005	30	CFU count	[167]
317					Gr+	*Bacillus subtilis*	no	0.005			
318		N-doped, aPDT			Gr−	*Escherichia coli*	yes	0.005	93		
319					Gr+	*Bacillus subtilis*	yes	0.005	94		
320	Carbon dots	antimicrobial agent-derived, N-doped	3.8	9.79	Gr−	*Escherichia coli*	yes	0.01	99	CFU count	[154]
321					Gr+	*Staphylococcus aureus*	yes	0.01	99		
322	Carbon dots	N-doped, aPDT	3		Gr−	*Escherichia coli*	yes	0.2	80	CFU count	[115]
323					Gr+	*Staphylococcus aureus*	yes	0.2	89		
324	Carbon dots	from citric acid	3.81	−22.09	Gr+	*Staphylococcus aureus*	yes	3.45	87	CFU count	[160]
325		from citric acid, aPDT					yes	3.45	99		
326	Carbon dots	antimicrobial agent-derived, N-doped	2.6	−7.5	Gr−	*Escherichia coli*	yes	0.25	100	CFU count	[69]
327					Gr−	*Klebsiella pneumoniae*	yes	1	100		
328					Gr+	*Staphylococcus aureus*	yes	1	100		
329					Gr+	*Staphylococcus epidermidis*	yes	0.25	100		
330	Carbon dots	antimicrobial agent-derived, N-doped	5	−39	Gr−	*Escherichia coli*	yes	5		ZOI	[96]
331					Gr+	*Staphylococcus aureus*	yes	5			
332	Carbon dots	N-doped	6		Fungi	*Cladosporium cladosporioides*	yes	0.05		images of damaged cells obtained by optical microscopy	[75]
333	Carbon dots	from D-glucose and sodium polyacrylate	1	−36	Fungi	*Candida albicans*	no	0.5		OD	[63]
334		from urea and citric acid, N-doped	2	−22			no	0.5			
335		D-glucosamine hydrochloride and 1,3-diaminobenzene, N-doped	2	14.3			yes	0.397	50		
336	Carbon dots	large antimicrobial agent-derived, N-doped	5.3		Gr−	*Escherichia coli*	yes	0.15	100	CFU count	[155]
337							yes	0.1	100	OD	
338					Gr+	*Staphylococcus aureus*	yes	0.1	100	CFU count	
339							yes	0.1	100	OD	
340		medium-sized antimicrobial agent-derived, N-doped	3.9		Gr−	*Escherichia coli*	yes	0.15	100	CFU count	
341							yes	0.1	100	OD	
342					Gr+	*Staphylococcus aureus*	yes	0.075	100	CFU count	
343							yes	0.075	100	OD	
344		Small, antimicrobial agent-derived, N-doped	2		Gr−	*Escherichia coli*	yes	0.075	100	OD	
345							yes	0.1	100	CFU count	
346					Gr+	*Staphylococcus aureus*	yes	0.05	100	CFU count	
347							yes	0.05	100	OD	
348	Carbon dots	antimicrobial agent-derived, N-doped			Gr−	*Escherichia coli*	yes	0.15	100	CFU count	[159]
349					Gr−	*Proteus vulgaris*	yes	0.15	100		
350					Gr−	*Pseudomonas aeruginosa*	yes	0.15	100		
351					Gr+	*Bacillus subtilis*	no	0.15			
352					Gr+	*Staphylococcus aureus*	no	0.15			
353	Carbon dots	N-doped	2.5	23	Gr+	*Staphylococcus aureus*	yes	0.256	100	OD	[120]
354					Gr+	*Staphylococcus epidermidis*	yes	0.256	100		
355	Carbon dots	N-doped	2		Fungi	*Pseudoperonospora cubensis*	yes	40	20	Mycological	[74]
356	Carbon dots	antimicrobial agent-derived, N-doped	13		Fungi	*Coriolus versicolor*	yes	0.6	100	Mycological	[4]
357					Fungi	*Gloeophyllum trabeum*	yes	0.84	100		
358	Carbon dots	N-doped	3.44	38.87	Gr−	*Escherichia coli*	yes	1.25	100	MTT staining intensity	[91]
359					Gr−	*Pseudomonas aeruginosa*	yes	0.15	100		
360					Gr+	*Bacillus subtilis*	yes	0.0156	100		
361					Gr+	*Staphylococcus aureus*	yes	0.0047	100		
362					Fungi	*Saccharomyces cerevisiae*	yes	0.3215	100		
363	Graphene	Graphite exfoliation	1350 ^7^	−17.7	Gr−	*Salmonella enterica*	yes	0.025	100	CFU count	[68]
364					Gr+	*Listeria monocytogenes*	yes	0.25	100		
365	Carbon nanofibers	methane decomposition over a 75Ni–15Cu–Al_2_O_3_ catalyst			Gr−	*Escherichia coli*	yes	0.1	100	CFU count	[84]
366					Gr+	*Staphylococcus aureus*	yes	0.1	100		
367		methane decomposition over a 90Ni–Al_2_O_3_ catalyst			Gr−	*Escherichia coli*	yes	0.1	100		
368					Gr+	*Staphylococcus aureus*	yes	0.1	100		
369	Nanobiochar	from date seeds			Gr−	*Escherichia coli*	no	0.0195		CFU count	[81]
370					Gr−	*Pseudomonas* *aeruginosa*	no	0.0195			
371					Gr+	*Staphylococcus aureus*	no	0.0195			
372					Gr+	*Staphylococcus epidermidis*	no	0.0195			
373	Nanobiochar	from crayfish shell			Gr−	*Escherichia coli*	no			ZOI	[108]

Notes: ^1^—Fullerenes are C_60_, unless otherwise specified; carbon dots are synthesized via the bottom-up approach, unless otherwise specified. ^2^—”size” denotes diameter for fullerenes and carbon dots, length for carbon nanotubes, and lateral size for graphene oxide and graphene. ^3^—Values are taken from [130]. ^4^—Value is taken from [257]. ^5^—Value is not explicitly specified; it is taken as the maximum value in the range used for other compounds. ^6^—LB medium is Luria–Bertani medium; MH medium is Mueller–Hinton medium. ^7^—Value is taken as the average of the given range. ^8^—Sizes were determined from electron microscopy images.

## 5. Conclusions

In summary, this review analyzes the antimicrobial (antibacterial and antifungal) activity of carbon nanomaterials based on published data, focusing on the five most studied types in this area, fullerenes, NDs, GO, CNTs, and CDs, primarily in their pristine form or at least without additional antimicrobial agents or active targeting. NDs appear to be the least toxic to microbes, with typical inhibitory concentrations of 0.016–50 mg/mL, whereas the other nanomaterials have typical values ranging from tens to several hundreds of μg/mL. The antimicrobial activity of fullerenes is significantly enhanced by cationic functionalization and by application in aPDT. For CNTs, antimicrobial activity correlates positively with decreasing length, and for CDs, high antimicrobial activity is associated with a positive zeta-potential. The moderate antimicrobial activity and detectable toxicity to animals and humans are counterbalanced by the fact that the toxicity is generally not acute, and the non-specific mechanisms of action cover a wide range of microorganisms while reducing the likelihood of resistance development. Carbon nanomaterials also show pronounced synergy with other antimicrobial agents and physical disinfection methods. Their antimicrobial properties can be valuable in various fields: medicine, food industry, wood processing, and water purification.

## Data Availability

No new data were created or analyzed in this study.

## Abbreviations

aPDT	Antimicrobial photodynamic therapy
CDs	Carbon dots
CFU	Colony-forming units
CLSI	Clinical and laboratory standards institute
CNTs	Carbon nanotubes
DLS	Dynamic light scattering
DNA	Deoxyribonucleic acid
GO	Graphene oxide
Gr–	Gram-negative
Gr+	Gram-positive
MBC	Minimal bactericidal concentration
MIC	Minimal inhibitory concentration
MWNTs	Multi-walled carbon nanotubes
NADH	β-nicotinamide adenine dinucleotide, reduced form
NDs	Nanodiamonds
OD	Optical density
ROS	Reactive oxygen species
SWNTs	Single-walled carbon nanotubes
SEM	Scanning electron microscopy
TEM	Transmission electron microscopy
ZOI	zone of inhibition
